# Oligomeric ricinoleic acid preparation promoted by an efficient and recoverable Brønsted acidic ionic liquid

**DOI:** 10.3762/bjoc.16.34

**Published:** 2020-03-10

**Authors:** Fei You, Xing He, Song Gao, Hong-Ru Li, Liang-Nian He

**Affiliations:** 1 State Key Laboratory and Institute of Elemento-Organic Chemistry, College of Chemistry, Nankai University, Tianjin 300071, China; 2 College of Pharmacy, Nankai University, Tianjin 300353, China

**Keywords:** bio-lubricant, ionic liquids, oligomeric ricinoleic acid, ricinoleic acid, sustainable catalysis

## Abstract

Raw material from biomass and green preparation processes are the two key features for the development of green products. As a bio-lubricant in metalworking fluids, estolides of ricinoleic acid are considered as the promising substitute to mineral oil with a favorable viscosity and viscosity index. Thus, an efficient and sustainable synthesis protocol is urgently needed to make the product really green. In this work, an environment-friendly Brønsted acidic ionic liquid (IL) 1-butanesulfonic acid diazabicyclo[5.4.0]undec-7-ene dihydrogen phosphate ([HSO_3_-BDBU]H_2_PO_4_) was developed as the efficient catalyst for the production of oligomeric ricinoleic acid from ricinoleic acid under solvent-free conditions. The reaction parameters containing reaction temperature, vacuum degree, amount of catalyst and reaction time were optimized and it was found that the reaction under the conditions of 190 °C and 50 kPa with 15 wt % of the [HSO_3_-BDBU]H_2_PO_4_ related to ricinoleic acid can afford a qualified product with an acid value of 51 mg KOH/g (which corresponds to the oligomerization degree of 4) after 6 h. Furthermore, the acid value of the product can be adjusted by regulating the reaction time, implying this protocol can serve as a versatile method to prepare the products with different oligomerization degree and different applications. The other merit of this protocol is the facile product separation by stratification and decantation ascribed to the immiscibility of the product and catalyst at room temperature. It is also worth mentioning that the IL catalyst can be used at least for five cycles with high catalytic activity. As a result, the protocol based on the IL catalyst, i.e. [HSO_3_-BDBU]H_2_PO_4_ shows great potential in industrial production of oligomeric ricinoleic acid from ricinoleic acid.

## Introduction

In recent years, the biomass has attracted much attention due to its abundance, renewability and potential of conversion to useful chemicals. It can’t be denied that the diverse transformation and utilization of biomass provides an alternative avenue to liberate us from the reliance on petroleum resource [1]. Nowadays, fuels, fine chemicals and functional molecules/materials can be derived from biomass such as lignocellulose and plant oils [2–3], wherein the plant oils play an important role in the polymer industry [4]. Especially, the characteristics of non-volatility and biodegradability make plant oil the most promising material to develop functional polymeric materials with superior performance [5–7].

Castor oil is one kind of nonedible oil and can be extracted from the seeds of the castor bean plant. Globally, around one million tons of castor seeds are produced every year and the leading producing areas are India, China, and Brazil [8]. The castor oil has long been used as purgative or laxative to counter constipation and nowadays it is used commercially as a highly renewable resource for the chemical industry [9–10]. Numerous platform chemicals such as ricinoleic acid and undecylenic acid can be prepared from castor oil, wherein ricinoleic acid is a crucial platform chemical for derivation of useful chemicals [11–14].

Ricinoleic acid can be easily prepared by hydrolysis of castor oil [15]. The presence of both hydroxy and carboxy groups in the molecule of ricinoleic acid enables it to undergo intermolecular esterification, thus resulting in the formation of the oligomeric ricinoleic acid. The oligomeric ricinoleic acid with different acid value (which is an indirect index for oligomerization degree) has different applications. For example, as an additive in shampoos, the oligomeric ricinoleic acid with an acid value of 60–90 mg KOH/g is required while for cosmetic formulations, an acid value of 20–40 mg KOH/g is suitable [16–19]. The oligomeric ricinoleic acid can also be used as lubricant for metal cutting oils due to its appropriate viscosity, good adsorptivity and film formation ability on metal surfaces. Furthermore, the biodegradability of these estolides in the environment makes them attractive as green products.

In parallel with the increasing demand for high-quality oligomeric ricinoleic acid, the synthetic method has kept on developing. Traditionally, *p*-toluenesulfonic or sulfuric acid are used as catalysts for the preparation of oligomeric ricinoleic acid. However, the equipment corrosion and the tedious workup process are inevitable, which reduce the process efficiency. Moreover, the byproduct formation and the resulting product coloration reduce the product quality. To address the above issues, the enzyme catalysis is proposed accordingly. Nevertheless, the high cost, low efficiency and operational unstability of enzymatic reaction make it difficult to industrial production [20–25]. Very recently, tin(II) 2-ethylhexanoate has been reported as an efficient catalyst for the synthesis of oligomeric ricinoleic acid [19]. Unfortunately, the separation of oligomeric ricinoleic acid and the recovery of the catalyst still encounters difficulties. Therefore, it is urgently desirable to develop an efficient, green and recyclable catalyst and design simple operating procedures for the preparation of estolides oligomeric ricinoleic acid.

Ionic liquids (ILs) have been proved to be efficient catalysts in various organic syntheses due to the designable structure, tunable properties as well as superior solubility [26–27]. Furthermore, the thermal stability and negligible vapor pressure of ILs can facilitate the product separation after reaction. To the best of our knowledge, the IL 1-butylsulfonic-3-methylimidazolium tosylate ([HSO_3_-BMim]TS) can be used as catalyst in the synthesis of oligomeric ricinoleic acid, the estolide with an acid value of 48 ± 2.5 mg KOH/g was obtained after 14 h [28]. Nevertheless, the product separation and catalyst reusability have not yet been investigated until now.

**Scheme 1:**
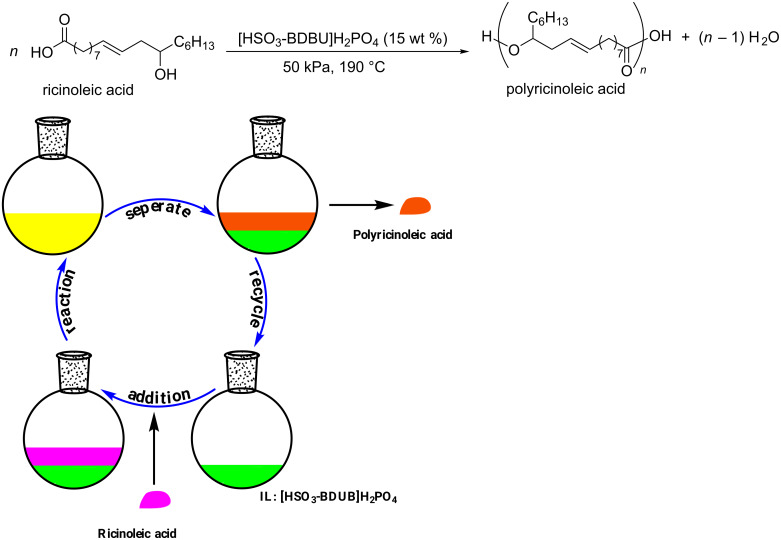
[HSO_3_-BDBU]H_2_PO_4_-promoted oligomerization and separation.

Based on the fact that Brønsted acids present excellent catalytic activity for this intermolecular esterification reaction and Brønsted acidic ionic liquids have been successfully used as catalyst in organic syntheses [29–31], we designed a series of Brønsted acidic ionic liquids and applied them as catalysts for the preparation of oligomeric ricinoleic acid from ricinoleic acid to develop a facile synthesis and separation protocol. Gratifyingly, the IL [HSO_3_-BDBU]H_2_PO_4_ showed to be efficient both in synthesis and product separation [32]. Through process parameter selection, it was found that a product with different acid value can be obtained by changing the reaction time at 190 °C and 50 kPa with 15 wt % IL as catalyst. The viscosity characterization showed the product derived from 6 h of reaction, whose acid value is 51 mg KOH/g and the corresponding average oligomerization degree is 4, meets the requirement of lubricant additive index. Notably, after reaction, the product and IL catalyst can be easily separated by phase separation and the recovered catalyst can be used at least for five cycles without obvious deactivation, which shows tremendous advantage for large-scale industrial application. The reaction and separation procedures are depicted in Scheme 1.

## Results and Discussion

**Scheme 2:**
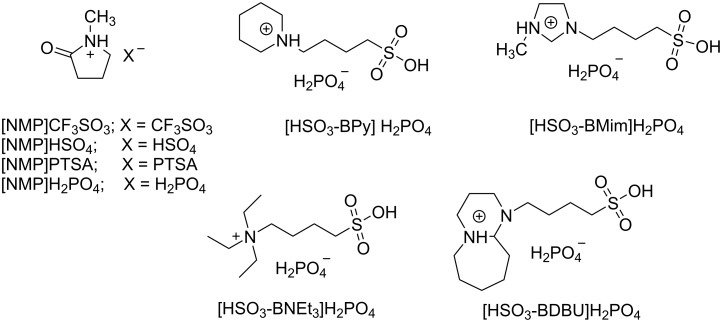
Structures of ILs used in this work.

Considering protic acids have catalytic effects on the esterification reaction, we designed and synthesized a series of ILs containing protic anions as shown in Scheme 2. With the synthesized ILs as catalyst, the ricinoleic acid esterification was performed and the catalytic activity of these functional ILs was evaluated with the acid value of the product as it is a convenient index to monitor the degree of oligomerization by reflecting the concentration of carboxy groups in the system, which has been cross verified by HPLC method [19,28].

The results showed that the protic anion is necessary for the intermolecular esterification as no catalytic activity can be observed for the IL with aprotic anion (Table 1, entries 1 and 2). For the ILs containing protic anions, the ILs containing polyprotic anions are more conducive to esterification (Table 1, entries 1–4 vs 5), which may originate from its higher proton concentration in the reaction system. With H_2_PO_4_^−^ as anion, the ILs containing different cations were then synthesized and their catalytic activity on the esterification reaction was investigated. The results (Table 1, entries 5–9) revealed that the cation also affected the catalytic activity of the IL. Taking the decrease of acid value as catalytic activity index, the ionic liquid 1-butanesulfonic acid triethylamine dihydrogen phosphate ([HSO_3_-BNEt_3_]H_2_PO_4_) performed best among the tested ionic liquids in this study (Table 1, entry 8). However, the IL [HSO_3_-BDBU]H_2_PO_4_ showed a much more attractive feature in product separation as stratification of product and catalyst was observed in the flask within a few minutes after reaction (Table 1, entry 9). Therefore, the IL [HSO_3_-BDBU]H_2_PO_4_ not only acts as an efficient catalyst but also provides a facile protocol for product and catalyst separation and thus avoids the workup procedure. In this context, [HSO_3_-BDBU]H_2_PO_4_ was selected as the suitable catalyst for further investigations.

**Table 1:** Screening of ILs in the esterification of ricinoleic acid^a^.

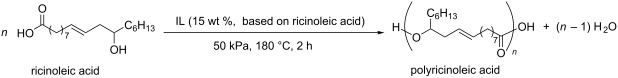		

Entry	IL	Acid value (mg KOH/g)

1	–	153
2	[NMP]CF_3_SO_3_	152
3	[NMP]HSO_4_	83
4	[NMP]PTSA	90
5	[NMP]H_2_PO_4_	68
6	[HSO_3_-BPy] H_2_PO_4_	63
7	[HSO_3_-BMim] H_2_PO_4_	53
8	[HSO_3_-BNEt_3_]H_2_PO_4_	51
9	[HSO_3_-BDBU]H_2_PO_4_	92

^a^Reactions were performed with ricinoleic acid (10 g, 30 mmol) and IL (15 wt %).

Having selected the IL [HSO_3_-BDBU]H_2_PO_4_ as catalyst, the process optimization was performed to further promote the intermolecular dehydration esterification. The reaction temperature, catalyst loading, and vacuum degree on the reaction outcome were in detail investigated and the acid value of the product was used to evaluate the reaction results (Table 2). It was easily found that a higher temperature was favorable for the formation of oligomeric ricinoleic acid (Table 2, entries 1–5). When the temperature was 190 °C, the acid value dropped to 88 mg KOH/g in 2 h. Further increasing the reaction temperature cannot lead to a significant drop of acid value. Thus, 190 °C was selected as the suitable reaction temperature. With the optimal reaction temperature, the amount of catalyst was studied at 190 °C and a vacuum degree of 50 kPa (Table 2, entries 6–9). As expected, the introduction of [HSO_3_-BDBU]H_2_PO_4_ can improve the intramolecular esterification greatly compared with the scenario without catalyst and the catalyst loading has a positive effect on the oligomerization degree. When the amount of [HSO_3_-BDBU]H_2_PO_4_ exceeded 15 wt % relative to ricinoleic acid, the catalytic efficiency was almost unchanged. Therefore, the optimum amount of catalyst was determined to be 15 wt % in the following investigation.

**Table 2:** Optimization of the key reaction parameters^a^.

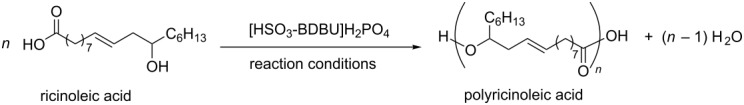				

Entry^b^	IL amount^c^ (wt %)	Temp. (°C)	Vacuum degree (kPa)	Acid value (mg KOH/g)

1	10	160	50	123
2	10	170	50	106
3	10	180	50	92
4	10	190	50	88
5	10	200	50	86
6	0	190	50	148
7	5	190	50	117
8	15	190	50	73
9	20	190	50	69
10	15	190	0	101
11	15	190	10	63
12	15	190	30	52
13	15	190	50	44
14	15	190	70	43

^a^Standard reaction conditions: ricinoleic acid (10 g, 30 mmol) and different amount of IL at a variety of temperature and vacuum degree; ^b^reaction time, 2 h (entries 1–9), 8 h (entries 10–14); ^c^based on ricinoleic acid.

The vacuum degree is another factor to affect the intermolecular esterification as different vacuum degree can result in a different water removal rate, which may lead to a different equilibrium toward estolides product. Five different levels of vacuum degrees were applied to the reaction system, the results showed the oligomerization degree increased with increasing vacuum degree (Table 2, entries 10–14). A stable acid value was approached when the vacuum degree was higher than 50 kPa. Consequently, 50 kPa was select as a suitable vacuum degree.

**Figure 1:**
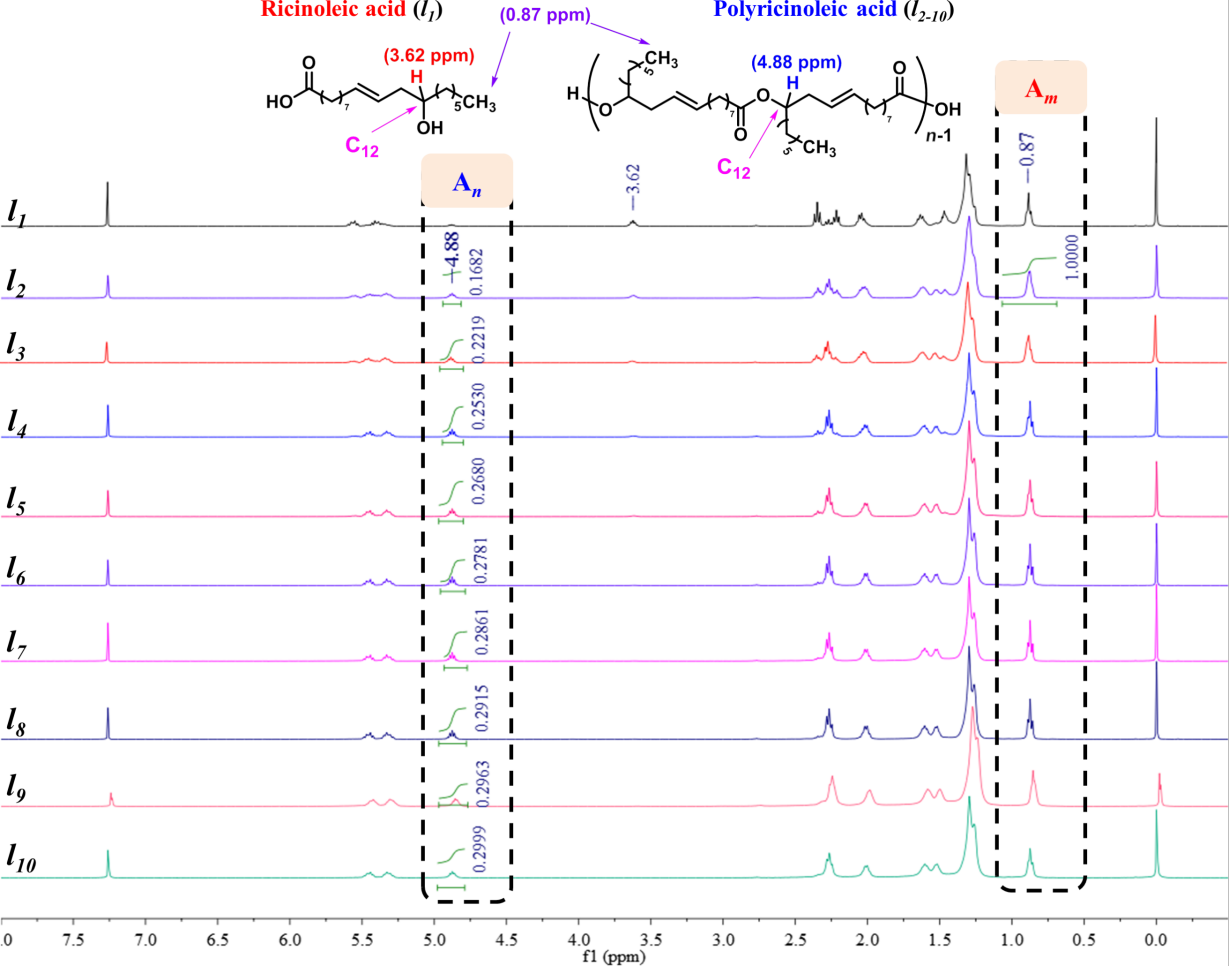
Monitoring oligomerization process by ^1^H NMR (400 MHz, CDCl_3_).

According to the above results, the suitable conditions for the esterification of ricinoleic acid were set at 190 °C under a vacuum degree of 50 kPa and with 15 wt % [HSO_3_-BDBU]H_2_PO_4_ as catalyst. Under the selected conditions, the acid value versus reaction time was inspected by sampling every 2 h (Table 3, entries 2–10). Simultaneously, the ^1^H NMR spectra of the samples in CDCl_3_ were also examined. In the ^1^H NMR study, a peak found at 3.62 ppm gradually disappeared and a new peak at 4.88 ppm appeared (Figure 1). This is associated with the changes of the chemical environment for the C_12_-H bond. That is, in ricinoleic acid (*l_1_*, Figure 1), the C_12_ is attached to the hydroxy group while in the corresponding ester product (*l_2–10_*, Figure 1), C_12_ is linked to the ester bond, thereby resulting in a change in the chemical shift of H connected with C_12_. As the chemical shift of H in methyl at 0.87 ppm does not change before and after the reaction, it is used as reference and the peak integral ratio of C_12_-H (A*_n_*) to methyl-H (A*_m_*) in the ^1^H NMR spectra is used to determine the oligomerization degree. Theoretically, oligomeric ricinoleic acids with different oligomerization degree have their characteristic A*_n_*/A*_m_* values as listed in Table 3. For a specific sample, both the peaks for C_12_-H and for methyl-H were integrated and the ratio of A*_n_*/A*_m_* was calculated. Then the resulting value was compared with the theoretical ones to determine the oligomerization degree of the product. It should be noted that this calculation is based on the assumption that the oligomerization degree of the product is monodisperse. Actually, the obtained oligomerization degree is the average oligomerization degree. By adopting the index A*_n_*/A*_m_*, the relationship of acid value and oligomerization degree can be constructed. According to the ^1^H NMR results, the value A*_n_*/A*_m_* increased as the reaction proceeded (Table 3, entries 2–10 vs entry 1), which means that the average oligomerization degree of oligomeric ricinoleic acid can be adjusted by changing the reaction time.

**Table 3:** Acid value and the average oligomerization degree of oligomeric ricinoleic acid versus reaction time^a^.

Entry	Reaction time (h)	Acid value (mg KOH/g)	Experimental A*_n_*/A*_m_*	Theoretical A*_n_*/A*_m_*	Average oligomerization degree

1	0	162	–	–	–
2	2	73	0.1682	0.1670	2
3	4	60	0.2219	0.2220	3
4	6	51	0.2530	0.2500	4
5	8	44	0.2680	0.2670	5
6	10	38	0.2781	0.2780	6
7	12	32	0.2861	0.2857	7
8	14	26	0.2915	0.2917	8
9	16	21	0.2963	0.2963	9
10	18	17	0.2999	0.3000	10

^a^The ricinoleic acid with an acid value of 162 mg KOH/g was used as raw material and interval sampling was performed every 2 h.

For oligomeric ricinoleic acid, its physicochemical properties depend on the oligomerization degree. In order to be used as lubriant in metal-working fluid, the viscosity and the viscosity index of oligomeric ricinoleic acid should fall into the acceptable scope according to Hostagliss L4 oil soluble lubricant additive product index. To determine the suitable reaction time and obtain the qualified product, the viscosity and viscosity index of samples derived from 4 h, 6 h and 8 h of reaction (i.e. NK-A, NK-B and NK-C) were further measured and the results are compared with the commercial product Hostagliss L4 (Table 4). As the results listed, NK-B meets the specifications of lubricant, which indicated that 6 h of reaction under the catalysis of [HSO_3_-BDBU]H_2_PO_4_ can afford the standard-compliant bio-lubricant oligomeric ricinoleic acid.

**Table 4:** Physicochemical properties of oligomeric ricinoleic acid compared to Hostagliss L4.

Entry	Sample	Acid value (mg KOH/g)	Viscosity (mm^2^/s)		Viscosity index

			40 °C	100 °C	

1	Hostagliss L4	52	400	45	169
2	NK-A	60	373	39	154
3	NK-B	51	408	46	171
4	NK-C	44	514	53	167

### Reusability of [HSO_3_-BDBU]H_2_PO_4_

**Figure 2:**
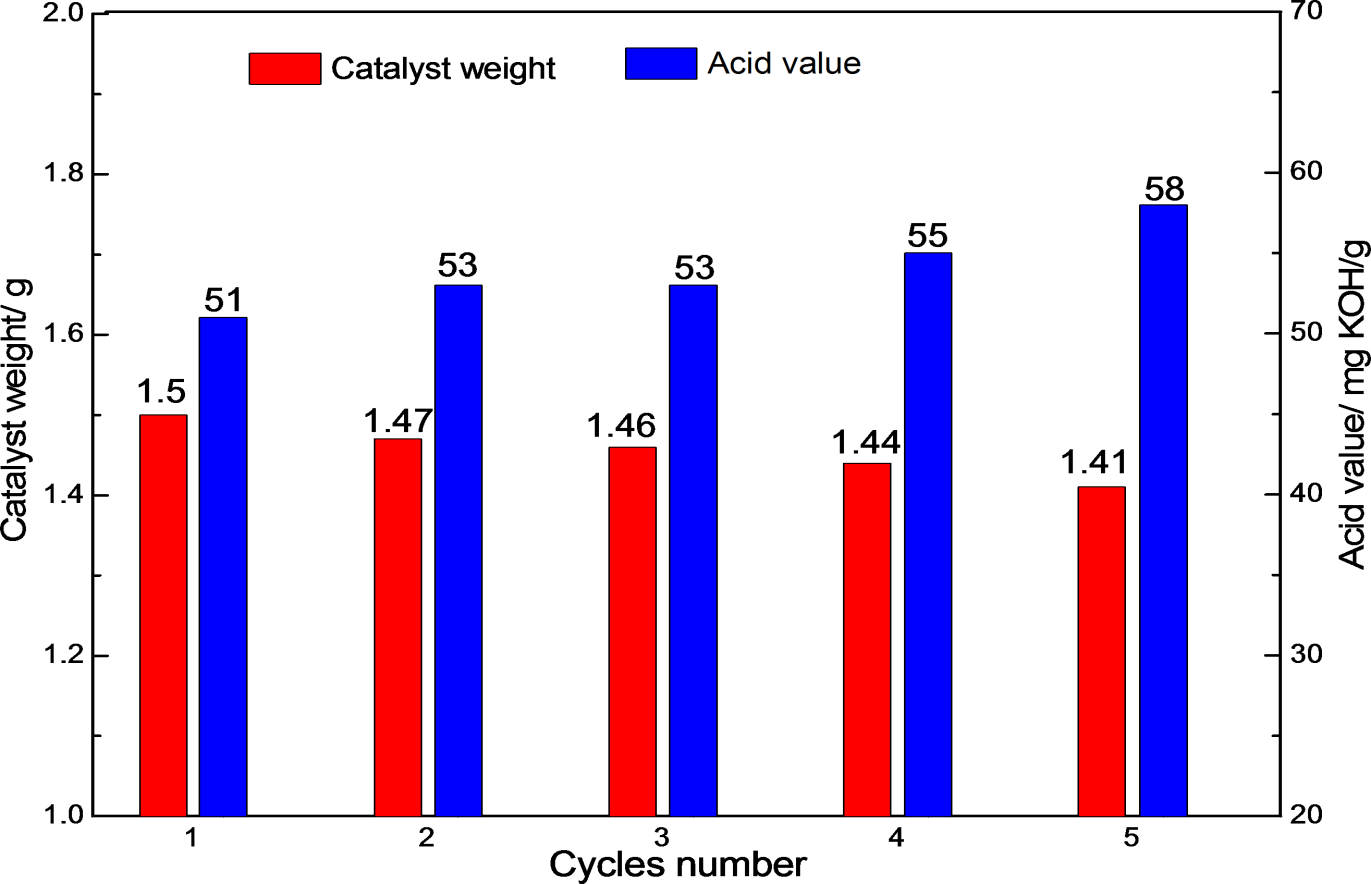
Reusability of the IL catalyst. Reaction conditions: 10 g (30 mmol) ricinoleic acid, 190 °C, 6 h, 50 kPa.

**Figure 3:**
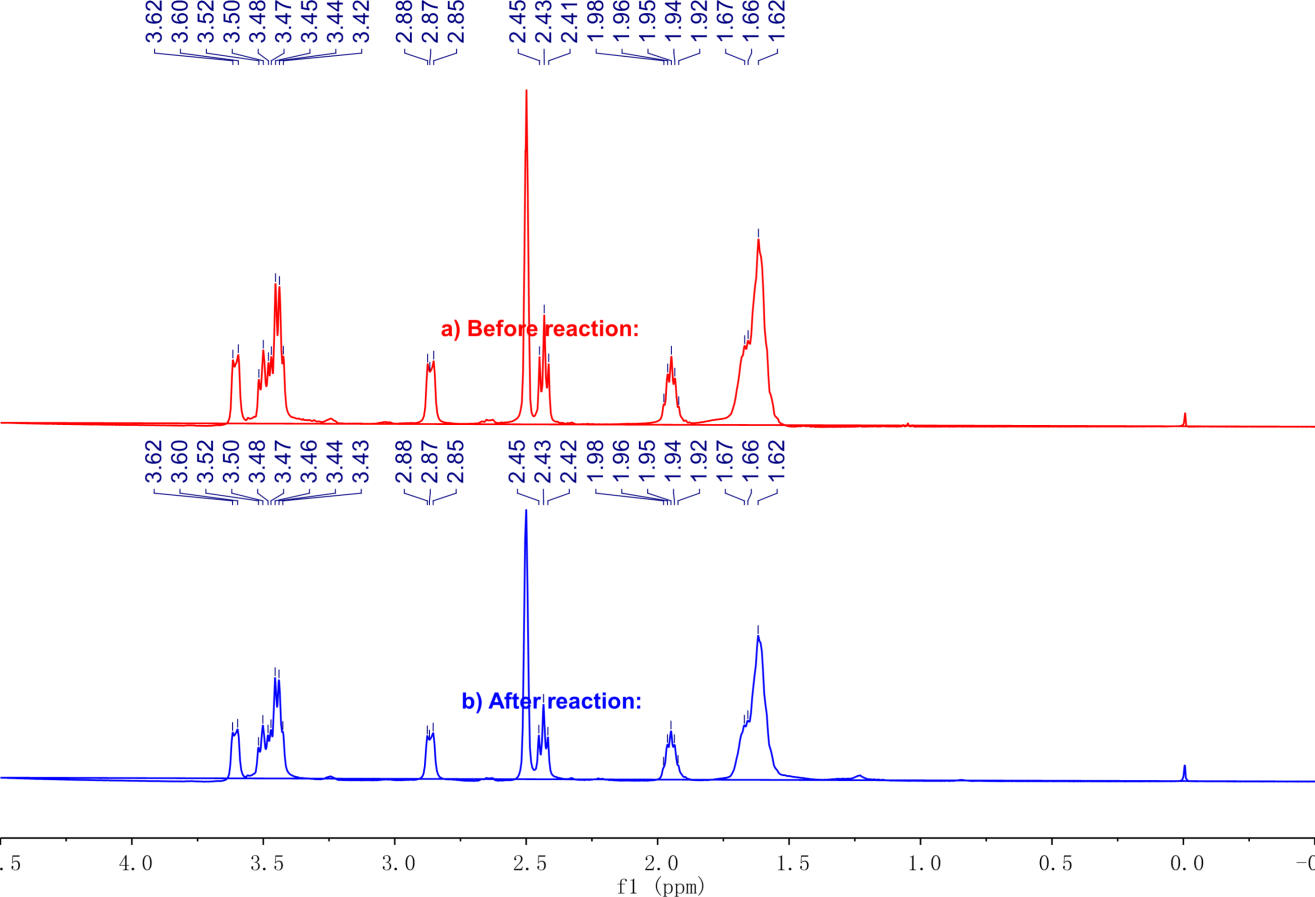
^1^H NMR (400 MHz, DMSO*-d*_6_) spectra of [HSO_3_-BDBU]H_2_PO_4_: a) Fresh one; b) used one after five cycles.

As the IL catalyst [HSO_3_-BDBU]H_2_PO_4_ is immiscible with the product at room temperature, stratification occurs after reaction, thus renders an easy catalyst recyclability and product separation. After decanting the upper product and washing the remaining IL with a small amount of dichloromethane, the weight of [HSO_3_-BDBU]H_2_PO_4_ was examined and then the fresh ricinoleic acid was added for the next cycle synthesis of oligomeric ricinoleic acid. The IL catalyst was used in five cycles of reaction and the resulting acid value of the product in each cycle was presented in Figure 2 along with the weight of catalyst. It can be seen that [HSO_3_-BDBU]H_2_PO_4_ can be utilized repeatedly for at least five times and only a slight decrease in catalytic activity was observed, which may be due to the weight loss of the recovered catalyst. To check the stability of the [HSO_3_-BDBU]H_2_PO_4_ during reusability, the ^1^H NMR of [HSO_3_-BDBU]H_2_PO_4_ was examined after five cycles of reaction and the results were presented in Figure 3. It can be seen that the IL catalyst was stable during the reaction as the spectrum remained basically unchanged compared with the fresh catalyst.

### Proposed mechanism

**Scheme 3:**
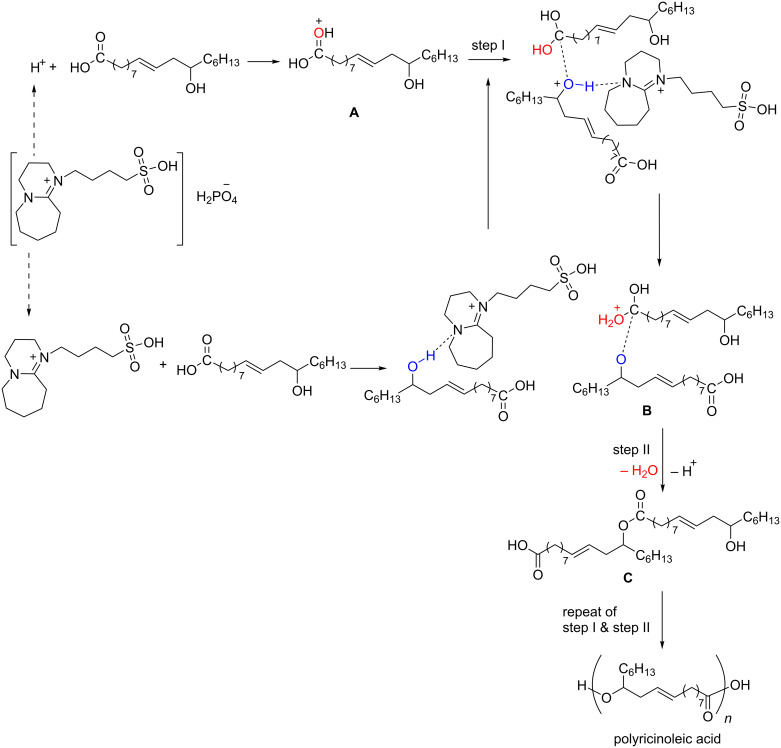
Proposed mechanism for [HSO_3_-BDBU]H_2_PO_4_ catalyzed oligomeric ricinoleic acid synthesis.

According to our experimental results, the protic acid is crucial for the reaction. Therefore, it is inferred that the reaction undergoes proton-promoted intermolecular esterification and the reaction mechanism with catalyst [HSO_3_-BDBU]H_2_PO_4_ is depicted in Scheme 3. Firstly, the Brønsted acidic IL [HSO_3_-BDBU]H_2_PO_4_ activates the carbonyl group of ricinoleic acid, leading to the generation of intermediate **A**. Concurrently, the hydroxy group in another ricinoleic acid molecule may be activated by the cation of IL and attacks the intermediate **A** (step I), generating a tetrahedral intermediate **B**. Finally, dehydration and deprotonation of the tetrahedral intermediate occurs (step II), forming dimeric ricinoleic acid **C**. The carboxyl and hydroxy groups in the dimeric ricinoleic acid may further undergo esterification, providing the oligomeric ricinoleic acid with different oligomerization degree.

## Conclusion

To conclude, a highly efficient Brønsted acidic IL catalyst [HSO_3_-BDBU]H_2_PO_4_ was developed for the esterification of ricinoleic acid. The reaction performed well under solvent-free conditions, the qualified biolubricant oligomeric ricinoleic acid can be prepared at 190 ºC and under vacuum degree of 50 kPa with 15 wt % IL as catalyst in 6 h. Both the acid value and average oligomerization degree of the product were determined and it was found the acid value was 51 mg KOH/g and the average oligomerization degree was 4. The reaction has excellent selectivity and no other byproducts were detected except water. More remarkably, a simple stratifying at room temperature will cause the separation of catalyst and product, which means the additional solvent extraction or distillation separation employed in the traditional IL catalysis was unnecessary in this system. Besides, the catalyst can be reused at least for five cycles without significant activity lost. Therefore, this protocol employing the Brønsted acidic ionic liquid as catalyst represents a green synthesis method for oligomeric ricinoleic acid and can be run in environmentally manner. It is a promising candidate for the commercial production of oligomeric ricinoleic acid from ricinoleic acid.

## Experimental

### Preparation procedure of [HSO_3_-BDBU]H_2_PO_4_

To prepare the IL [HSO_3_-BDBU]H_2_PO_4_, 0.1 mol (13.6 g) 1,4-butane sultone was mixed with 0.1 mol (15.2 g) 1,8-diazobicyclo[5,4,0]undec-7-ene (DBU) in a flask containing 50 mL of acetonitrile. After 24 h reflux at 80 °C, the reaction mixture was cooled to room temperature. Then 30 mL diethyl ether was added to the reaction mixture to precipitate the product. After that, the precipitate was collected by filtration and washed twice with diethyl ether. The resulting light yellow precipitate was then dried in vacuum at 60 ºC for 4 h. Afterwards, the aqueous solution containing a stoichiometric amount of phosphoric acid was added dropwise to 50 mL CH_2_Cl_2_ containing 0.05 mol (14.4 g) [HSO_3_-BDBU] and stirred at 60 °C for 4 h, forming a viscous liquid on the surface of CH_2_Cl_2_ which can be easily separated by centrifugation. Then the viscous liquid was washed twice with CH_2_Cl_2_ and dried at 100 ºC for 12 h, obtaining 18.6 g yellow viscous liquid with the yield of 96%. The resulting compound was identified to be IL [HSO_3_-BDBU]H_2_PO_4_. ^1^H NMR (400 MHz, DMSO*-d*_6_) δ 1.62–1.67 (m, 10H), 1.92–1.98 (m, 2H), 2.41–2.45 (t, *J* = 6.8 Hz, 2H), 2.85–2.88 (t, *J* = 4.8, 2H), 3.42–3.62 (m, 8H) ppm; ^13^C NMR (100 MHz, DMSO*-d*_6_) δ 19.65, 22.13, 22.88, 25.55, 27.12, 27.22, 27.90, 46.58, 48.52, 50.65, 52.92, 53.98, 165.98 ppm; FTIR (KBr) *ν*_max_/cm^−1^: 3317.18, 2939.11, 2867.62, 1621.52, 1527.51, 1452.10, 1328.75, 1201.00, 998.74, 726.90, 600.28; ESIMS (+) *m*/*z*: 100.1, 102.1, 153.2, 289.3, 390.1.

For the preparation of other ILs in this paper and for full experimental data see Supporting Information File 1.

### Catalytic dehydration esterification of ricinoleic acid and catalyst recycling

The dehydration esterification of ricinoleic acid was investigated using ILs as catalyst. In a typical run, 10 g (30 mmol) ricinoleic acid and 1 g (2.6 mmol) [HSO_3_-BDBU]H_2_PO_4_ were added into a 100 mL glass flask equipped with magnetic stirrer, a reflux condenser and connected with vacuum line. Then, under a stirrer rate of 500 r/min, the temperature was increased to 190 °C to promote the esterification reaction. Simultaneously, the reaction run at 50 kPa to remove the generated water. After 2 h of reaction, the reaction mixture was cooled to room temperature for stratifying. The supernatant was oligomeric ricinoleic acid and it could be decanted directly for further treatment and acid value analysis. The IL catalyst deposited in the lower layer can be washed with a small amount of dichloromethane to remove the residual oligoester. After that, the fresh ricinoleic acid was added for the second run reaction and the recovered catalyst can be used repeatedly.

### Product characterization

The oligomeric ricinoleic acid is a yellow oily liquid, and all experiments have yields greater than 90%, which are determined by weighing. The spectral results identified the product. ^1^H NMR (400 MHz, CDCl_3_) δ 0.85–0.88 (t, *J* = 3.9 Hz, 3H), 1.26–1.29 (m, 16H), 1.51–1.60 (m, 4H), 2.00–2.01 (m, 2H), 2.25–2.26 (m, 4H), 4.86–4.89 (m, 1H), 5.30–5.46 (m, 2H) ppm; ^13^C NMR (100 MHz, CDCl_3_) δ 14.08, 22.58, 25.11, 25.35, 27.20–27.35, 29.03–29.71, 31.75, 31.98, 33.62, 34.65, 73.69, 124.30, 132.51, 173.58 ppm; FTIR (KBr) *ν*_max_/cm^−1^: 3416.44, 3010.55, 2927.89, 2855.81, 1733.38, 1711.66, 1464.22, 1245.41, 1183.74, 725.11; ESIMS (+) *m*/*z*: 579.3, 876.6, 1139.7, 1437.8, 1716.9, 1997.1.

### Acid value determination

The acid value of product was determined using a modified ASTM D664 method [33], in which about 1 g sample was dissolved in 30 mL CH_2_Cl_2_ and the resulting solution was titrated against standard 0.1 N isopropanol KOH. Acid value (mg KOH/g sample) was then calculated as follows:


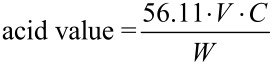


where *V* (mL) and *C* (mol/L) are the consumed volume and concentration of KOH solution, respectively, *W* (g) is the weight of the sample.

To exclude the presence of IL in supernatant and ensure the reliability of acid value in oligomerization degree determination, additional treatment was performed for the supernatant before acid value determination. That is, the supernatant was diluted with dichloromethane, and the putative IL in the sample was extracted three times with distilled water. Then the remaining organic phase was dried with Na_2_SO_4_ for 24 h followed by filtration. After that, the filtrate was collected and the dichloromethane solvent was removed by vacuum evaporation to obtain oligomeric ricinoleic acid for acid value measurement (Table 5, entries 1, 3 and 5). On the other hand, the acid value of supernatant without any treatment was also determined for comparison (Table 5, entries 2, 4 and 6). The acid values of the two treatments were identical, indicating that IL settled well after the reaction and was absent in the product.

**Table 5:** Acid value measurement results of two different treatments for the supernatant.

Entry^a^	Reaction time (h)	Acid value (mg KOH/g)

1	1	108
2	1	108
3	2	74
4	2	73
5	4	60
6	4	60

^a^Reaction conditions: 10 g (30 mmol) ricinoleic acid, 1 g (2.6 mmol) [HSO_3_-BDBU]H_2_PO_4_, 190 °C, 50 kPa.

## Supporting Information

File 1 Experimental part and spectra of synthesized compounds.
