# Supplementary material for: Oligomeric ricinoleic acid preparation promoted by an efficient and recoverable Brønsted acidic ionic liquid
Source: Beilstein J Org Chem. 2020 Mar 10;16:351–61. doi: 10.3762/bjoc.16.34 (PMC7082613; doi:10.3762/bjoc.16.34)
Supplement: File 1 — Experimental part and spectra of synthesized compounds. [file Beilstein_J_Org_Chem-16-351-s001.pdf]

BEILSTEIN JOURNAL OF ORGANIC CHEMISTRY

Supporting Information

for

Oligomeric ricinoleic acid preparation promoted by an efficient and recoverable Brønsted acidic ionic liquid

Fei You, Xing He, Song Gao, Hong-Ru Li and Liang-Nian He

Beilstein J. Org. Chem. **2020,** *16,* 351–361. doi:10.3762/bjoc.16.34

Experimental part and spectra of synthesized compounds

License and Terms: This is a supporting information file under the terms of the Creative Commons Attribution License (https://creativecommons.org/licenses/by/4.0). Please note that the reuse, redistribution and reproduction in particular requires that the authors and source are credited.
The license is subject to the *Beilstein Journal of Organic Chemistry* terms and conditions: (https://www.beilstein-journals.org/bjoc)

Contents:

S3: General information

S3: General procedures for the preparation of ILs

S7: Characterization of ILs

S31: Products characterization

S35: References

General information

All chemicals in experiments were obtained commercially and used as received directly. The FT-IR spectra of the samples were recorded using Bruker Tensor 27 FT-IR in the range of 4000-400 cm^{-1} with KBr pellets. ^{1}H NMR spectra was recorded on Bruker AVANCE III HD 400 spectrometer (Bruker BioSpin, Rheinstetten, Germany) using $CDCl_3$, D_2O or DMSO-d_6 as solvent referenced to $CDCl_3$ (7.26 ppm), D_2O (4.79 ppm) or DMSO-d_6 (2.50 ppm). ^{13}C NMR spectra was recorded at 100 MHz in $CDCl_3$ (77.16 ppm), D_2O or DMSO-d_6 (39.52 ppm). Acid value analysis was performed on DZY-ZJ5 microcomputer acid value tester (Instruments And Meters Co., Ltd, Dalian, China). Viscosity analysis was performed on kinematic viscosity tester (Instruments And Meters Co., Ltd, Dalian, China) adopting ASTM D 445 standards. Melting points were determined on an XT4A melting point apparatus without calibration. Electrospray ionization mass spectra (ESI-MS) were recorded with an Agilent 6520 Q-TOF mass spectrometer.

General procedures for the preparation of ILs

[NMP]H_2PO_4: To prepare the IL [NMP]H_2PO_4, a dropwise addition of 1 equiv. of phosphoric acid to cooled N-methyl-2-pyrrolidinone was performed. After the addition, the reaction mixture was stirred for 24 h at 80 ºC. Then the water was removed by heating the residue at 80 ºC in high vacuum until the weight of the residue remained constant [1].

[NMP]HSO_4: To prepare the IL [NMP]HSO_4, 1 equiv. of sulfuric acid was added to cooled N-methyl-2-pyrrolidinone dropwise. Afterward, the resulting mixture was stirred for 24 h at 80 ºC. Then the water was removed by heating the residue at 80 ºC in high vacuum until the weight of the residue remained constant [1].

[NMP]PTSA: To prepare the IL [NMP]H_2PO_4, a dropwise addition of 1 equiv. of *p*-toluenesulfonic acid to cooled N-methyl-2-pyrrolidinone was performed. After the addition, the reaction mixture was stirred for 24 h at 80 ºC. Then the water was removed by heating the residue at 80 ºC in high vacuum until the weight of the residue remained constant [1].

[NMP]CF_3SO_3: To prepare the IL [NMP]H_2PO_4, 1 equiv. of trifluoromethanesulfonic acid was added to cooled N-methyl-2-pyrrolidinone dropwise. After the addition, the reaction mixture was stirred for 24 h at 80 ºC. Then the water was removed by heating the residue at 80 ºC in high vacuum until the weight of the residue remained constant.

[HSO_3-BDBU]H_2PO_4: To prepare the IL [HSO_3-BDBU]H_2PO_4, HSO_3-BDBU was prepared first. In the preparation of HSO_3-BDBU, 0.1 mol (13.6 g) 1,4-butane sultone was mixed with 0.1 mol (15.2 g) 1,8-diazobicyclo[5,4,0]undec-7-ene (DBU) in a flask containing 50 mL of acetonitrile. After 24 h of reflux at 80 ºC, the reaction mixture was cooled to room temperature. Then 30 mL diethyl ether was added to the reaction mixture to precipitate the product HSO_3-BDBU. After that, the precipitate was collected by filtration and washed twice with diethyl ether. The resulting light yellow precipitate was then dried in vacuum at 60 ºC for 4 h. Afterward, the CH_2Cl_2 solution of HSO_3-BDBU was prepared by dissolving 0.05 mol (14.4 g) HSO_3-BDBU in 50 mL CH_2Cl_2. Then, the aqueous solution containing a stoichiometric amount of phosphoric acid was added dropwise to the CH_2Cl_2 solution containing HSO_3-BDBU and stirred at 60 °C for 4 h, forming viscous liquid on the surface of CH_2Cl_2 which can be easily separated by centrifugation. Then the viscous liquid was washed twice with CH_2Cl_2 and dried at 100 ºC for 12 h, obtaining 18.6 g yellow viscous liquid with the yield of 96%. The resulting compound was identified to be IL [HSO_3-BDBU]H_2PO_4.

[HSO_3-BPy]H_2PO_4: To prepare the IL [HSO_3-BPy]H_2PO_4, HSO_3-BPy was prepared first. To prepare HSO_3-BPy, 0.1 mol (13.6 g) 1,4-butane sultone was mixed with 0.1 mol (7.9 g) pyridine in a flask containing 50 mL of acetonitrile. After 24 h of reflux at 80 ºC, the reaction mixture was cooled to room temperature and a precipitate was observed. The precipitate was collected by filtration and washed twice with diethyl ether. The resulting white precipitate was then dried in vacuum at 60 ºC for 4 h and HSO_3-BPy was obtained. Afterward, the aqueous solution containing a stoichiometric amount of phosphoric acid was added dropwise to 50 mL CH_2Cl_2 containing 0.05 mol (10.8 g) HSO_3-BPy and stirred at 60 °C for 4 h. The upper viscous liquid is then separated and washed twice with ether. Drying at 100 ºC for 12 h yielded a colorless viscous liquid (14.8 g) of 94%. The resulting compound was identified to be IL [HSO_3-BPy]H_2PO_4.

[HSO_3-BMim]H_2PO_4: To prepare the IL [HSO_3-BMim]H_2PO_4, HSO_3-BMim was prepared first. In the preparation of HSO_3-BMim, 0.1 mol (13.6 g) 1,4-butane sultone was mixed with 0.1 mol (8.2 g) methylimidazole in a flask containing 50 mL of acetonitrile. After 24 h of reflux at 80 ºC, the reaction mixture was cooled to room temperature and a precipitate was observed. The precipitate was collected by filtration and washed twice with diethyl ether. The resulting white precipitate was then dried in vacuum at 60 ºC for 4 h and HSO_3-BMim was obtained. Afterward, the aqueous solution containing a stoichiometric amount of phosphoric acid was added dropwise to 50 mL CH_2Cl_2 containing 0.05 mol (10.9 g) HSO_3-BMim and stirred at 60 °C for 4 h. The upper viscous liquid is then separated and washed twice with ether. Drying at 100 ºC for 12 h yielded a colorless viscous liquid (15.2 g) of 96%. The resulting compound was identified to be IL [HSO_3-BMim]H_2PO_4 [2].

[HSO_3-BNEt$_3$]H_2PO_4: To prepare the IL [HSO_3-BNEt$_3$]H_2PO_4, HSO_3-BNEt$_3$ was prepared first. To get HSO_3-BNEt$_3$, 0.1 mol (13.6 g) 1,4-butane sultone was mixed with 0.1 mol (10.1

g) triethylamine in a flask containing 50 mL of acetonitrile. After 24 h reflux at 80 ºC, the reaction mixture was cooled to room temperature and a precipitate was observed. The precipitate was collected by filtration and washed twice with diethyl ether. The resulting white precipitate was then dried in vacuum at 60 ºC for 4 h, affording HSO_3-$BNEt_3$. Afterward, the aqueous solution containing a stoichiometric amount of phosphoric acid was added dropwise to 50 mL CH_2Cl_2 containing 0.05 mol (11.9 g) HSO_3-$BNEt_3$ and stirred at 60 °C for 4 h. The upper viscous liquid is then separated and washed twice with ether. Drying at 100 ºC for 12 h yielded a colorless viscous liquid (16.0 g) of 95% . The resulting compound was identified to be IL [HSO_3-$BNEt_3$]H_2PO_4.

Characterization of ILs

[NMP]H_2PO_4:

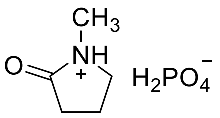

Colorless viscous liquid; ^{1}H NMR (400 MHz, $CDCl_3$) δ: 1.98-2.06 (m, 2H), 2.36-2.40 (t, J = 8.1 Hz, 2H), 2.84 (s, 3H), 2.36-3.40 (t, J = 7.1 Hz, 2H) ppm; ^{13}C NMR (100 MHz, $CDCl_3$) δ: 17.62, 29.53, 30.66, 49.39, 175.02 ppm; FT-IR (KBr) Vmax/ cm^{-1}: 2937.04, 2892.25, 2306.23, 1629.84, 1117.27, 1004.48, 882.52, 484.05; ESI-MS: m/z (+) 100.1, 198.9.

^{1}H NMR spectrum of [NMP]H_2PO_4:

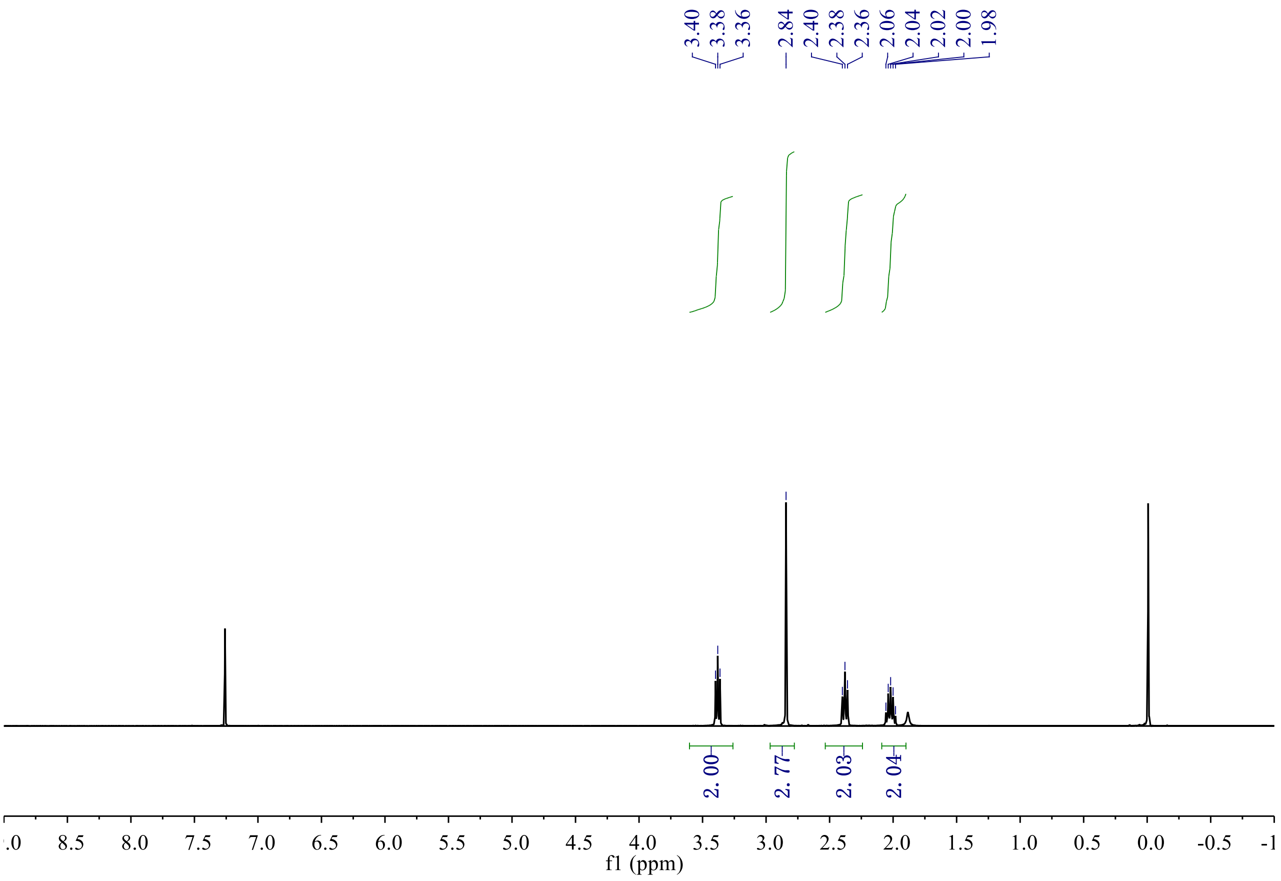

^{13}C NMR spectrum of [NMP]H_2PO_4:

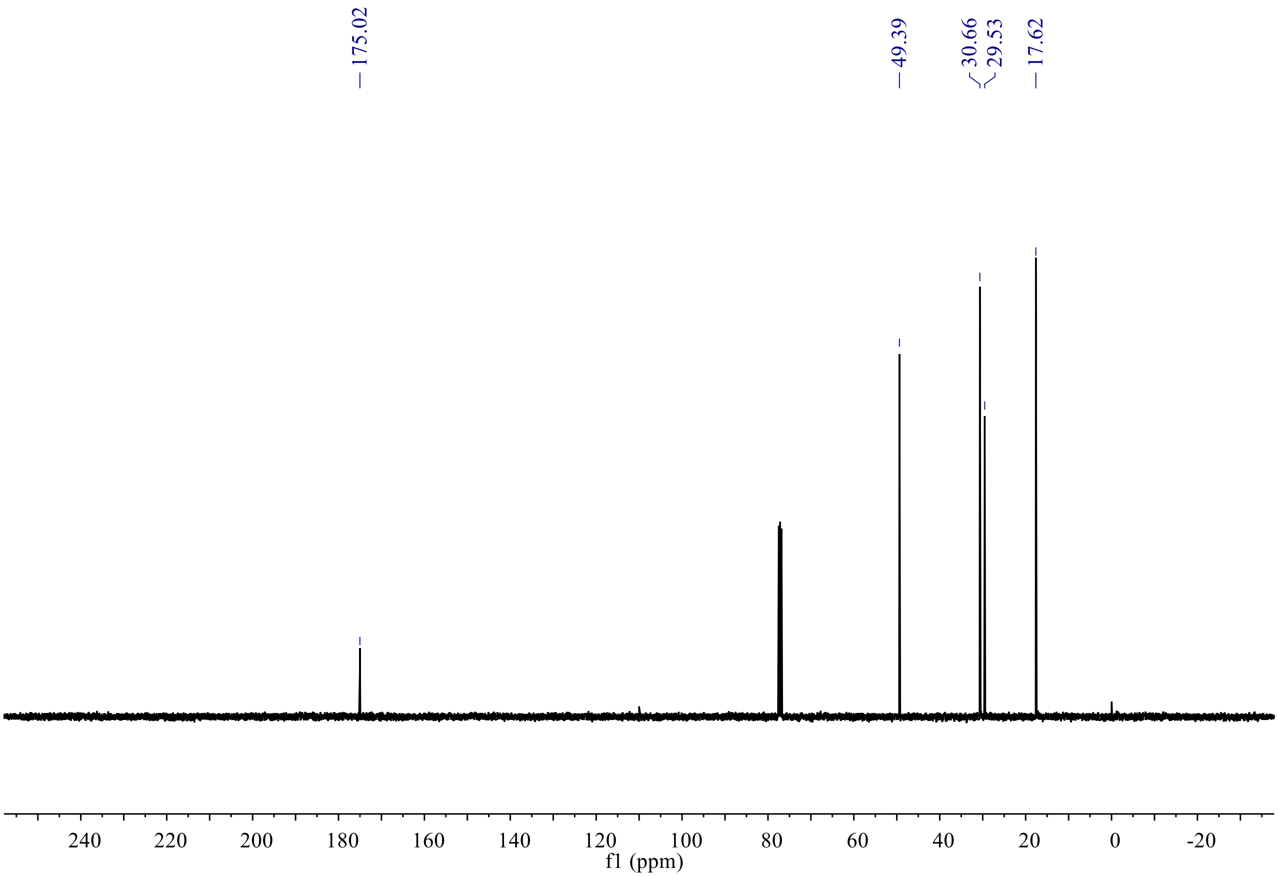

FT-IR spectrum of $[NMP]H_2PO_4$:

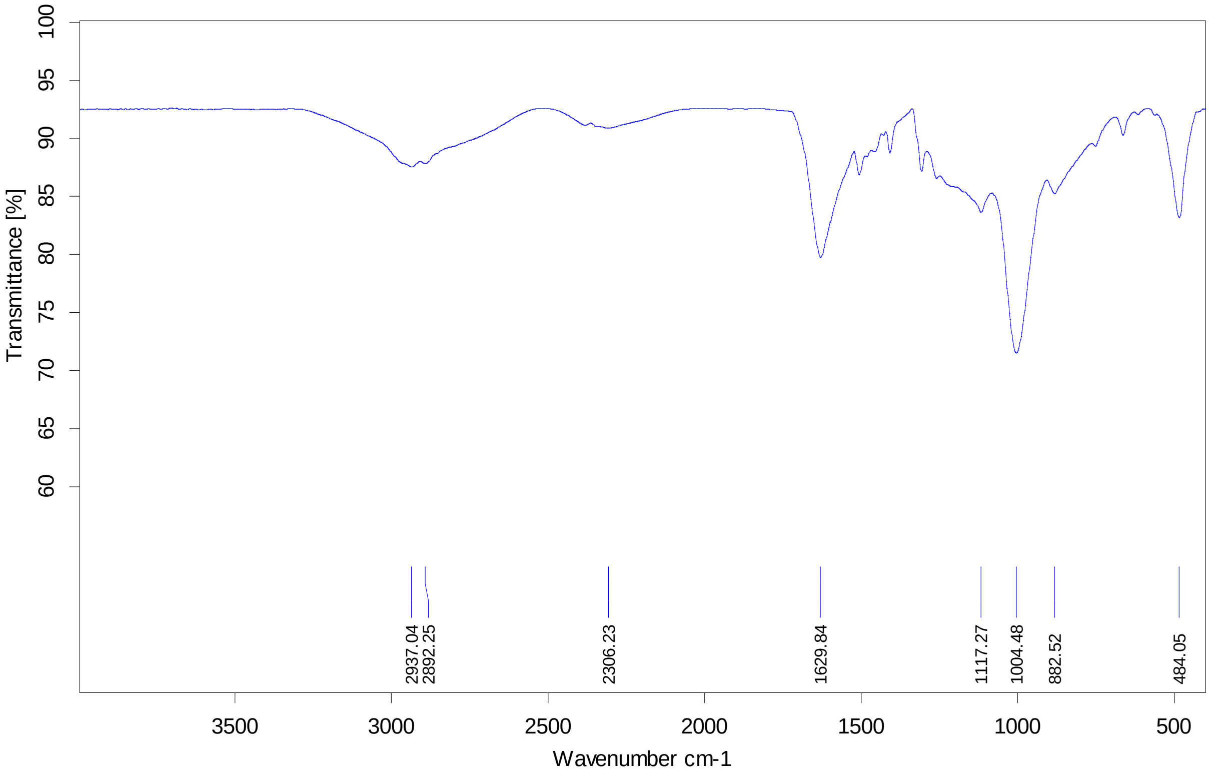

ESI-MS spectrum of $[NMP]H_2PO_4$:

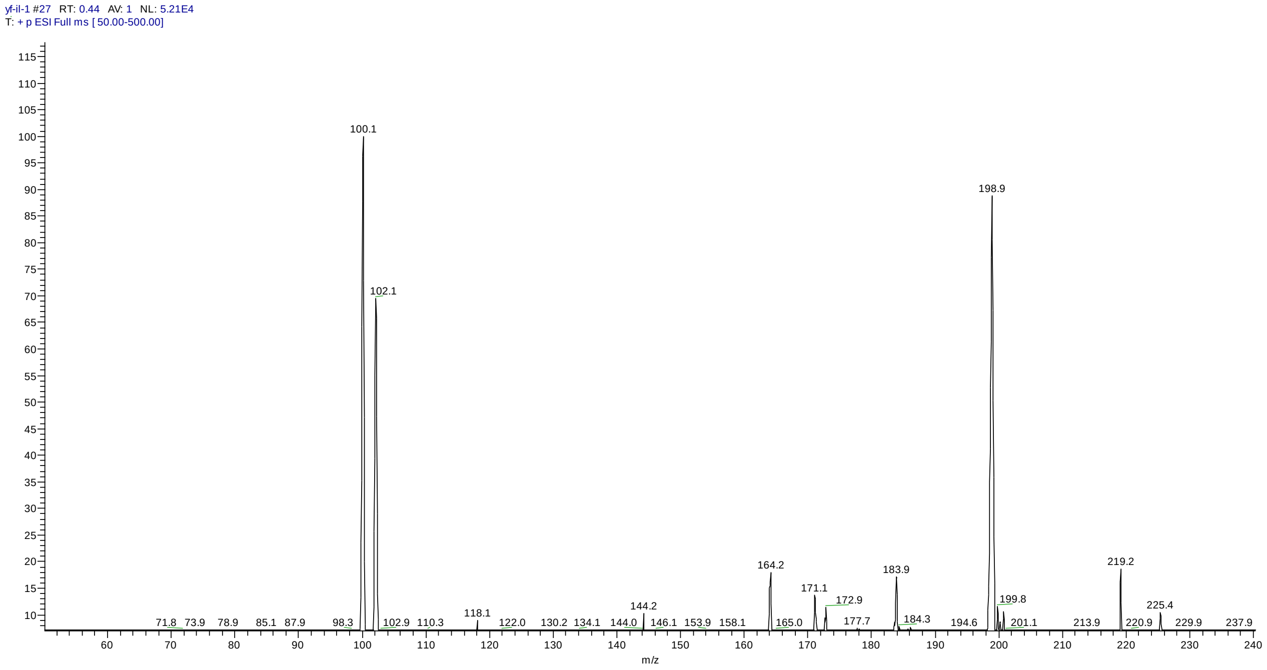

[NMP]HSO4:

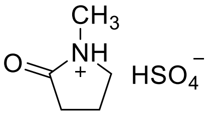

Colorless viscous liquid; ^{1}H NMR (400 MHz, $CDCl_3$) δ: 1.99-2.06 (m, 2H), 2.40-2.44 (t, J = 8.1 Hz, 2H), 2.85 (s, 3H), 3.37-3.41 (t, J = 7.1 Hz, 2H) ppm; ^{13}C NMR (100 MHz, $CDCl_3$) δ: 17.70, 29.83, 30.69, 49.76, 178.23 ppm; FT-IR (KBr) Vmax/ cm^{-1}: 3385.84, 3187.78, 2950.81, 1659.05, 1485.88, 1219.03, 1192.67, 1079.02, 1049.26, 885.08, 764.82, 590.62; ESI-MS: m/z (+) 100.1, 199.0.

^{1}H NMR spectrum of [NMP]HSO4:

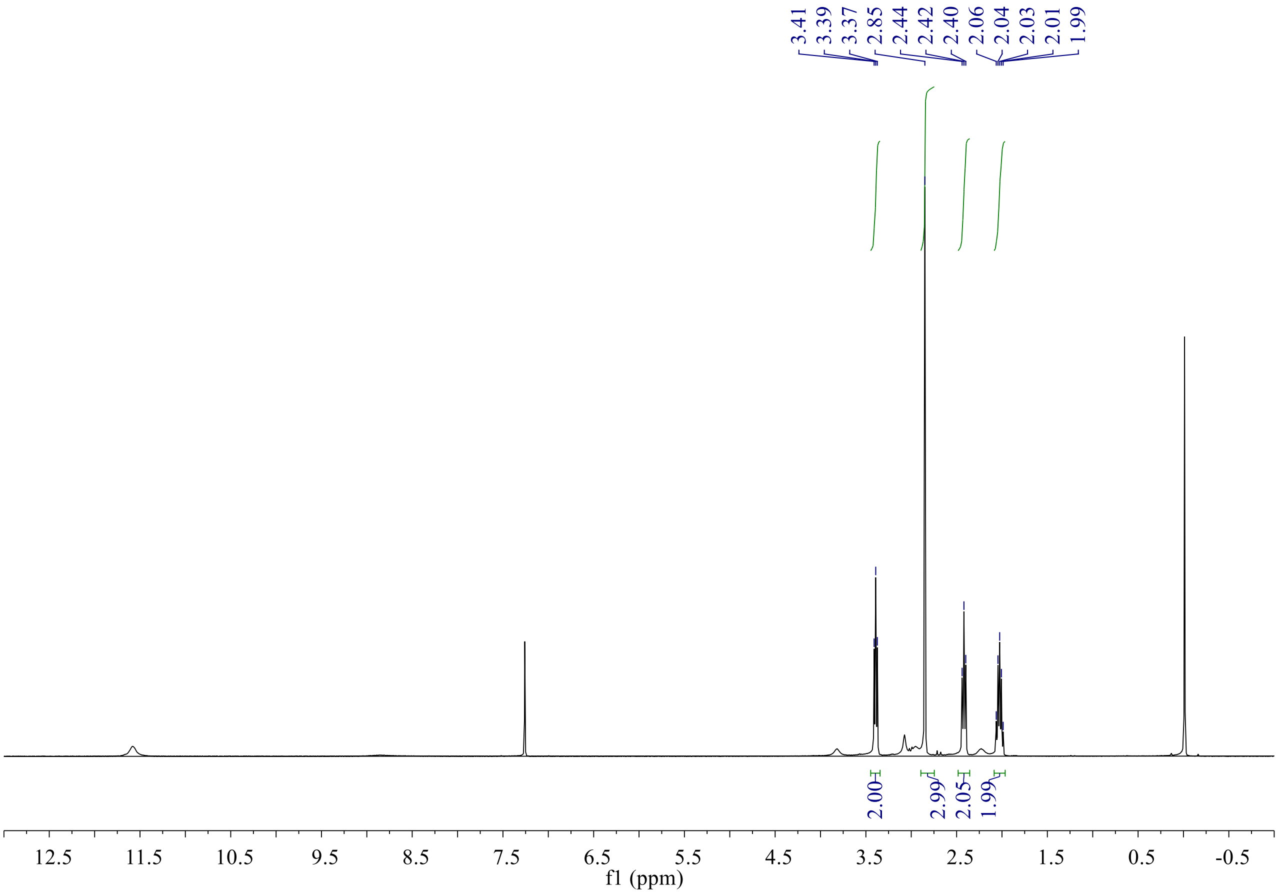

^{13}C NMR spectrum of [NMP]HSO_4:

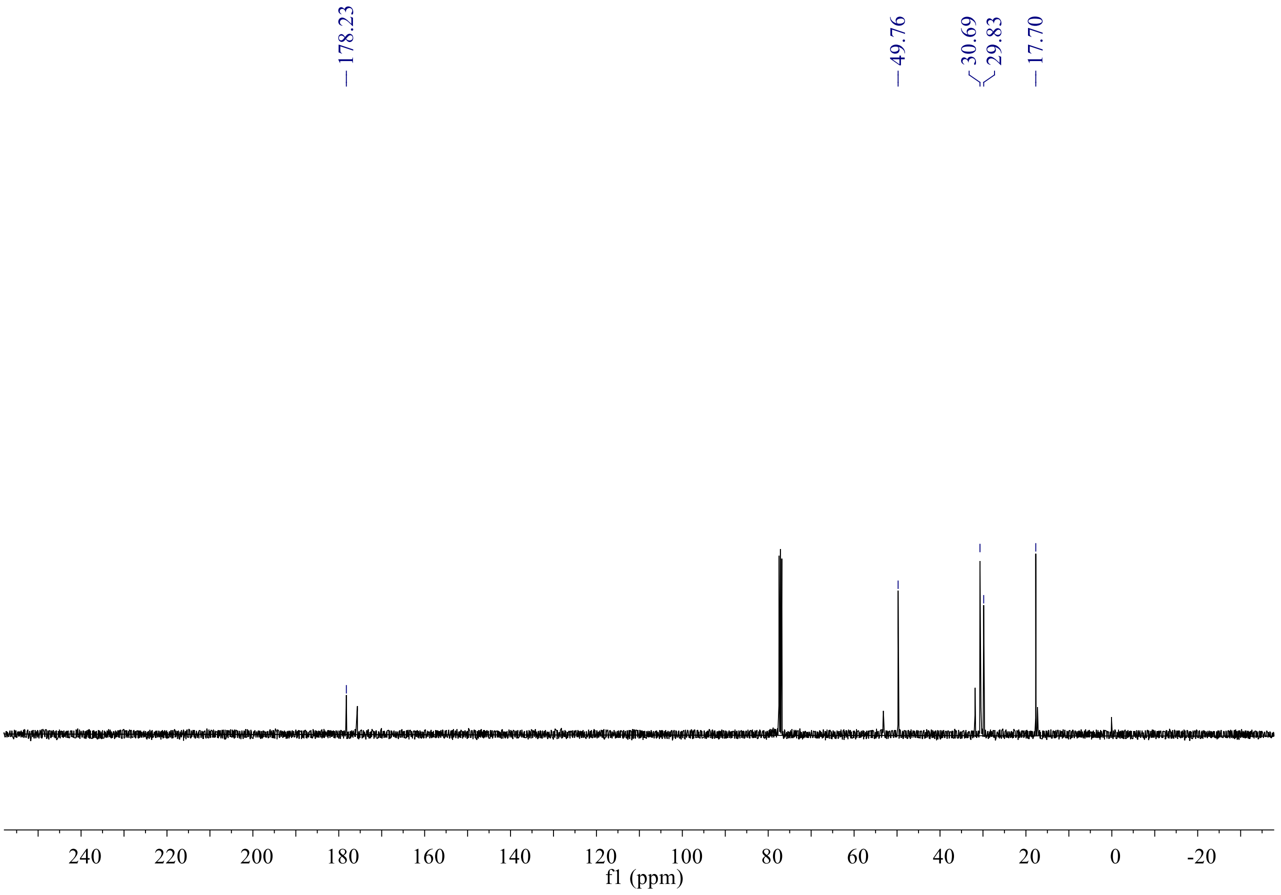

FT-IR spectrum of [NMP]HSO_4:

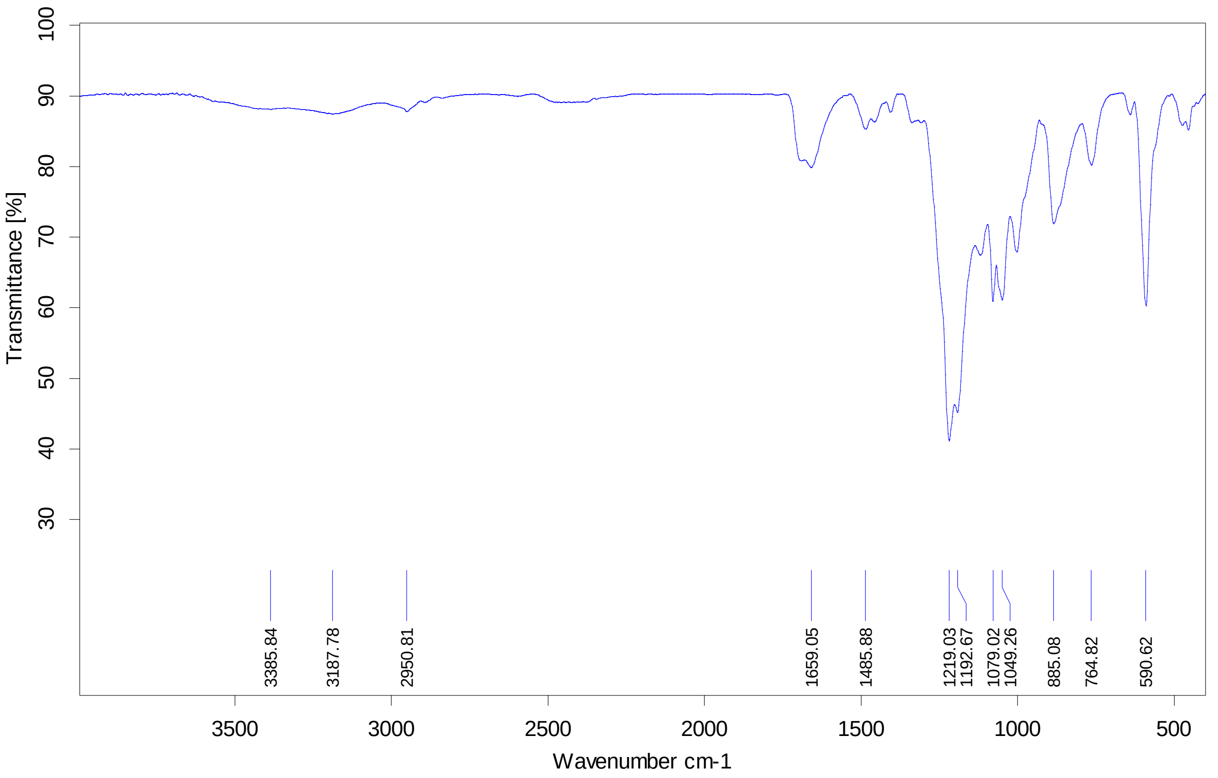

ESI-MS spectrum of [NMP]HSO_4:

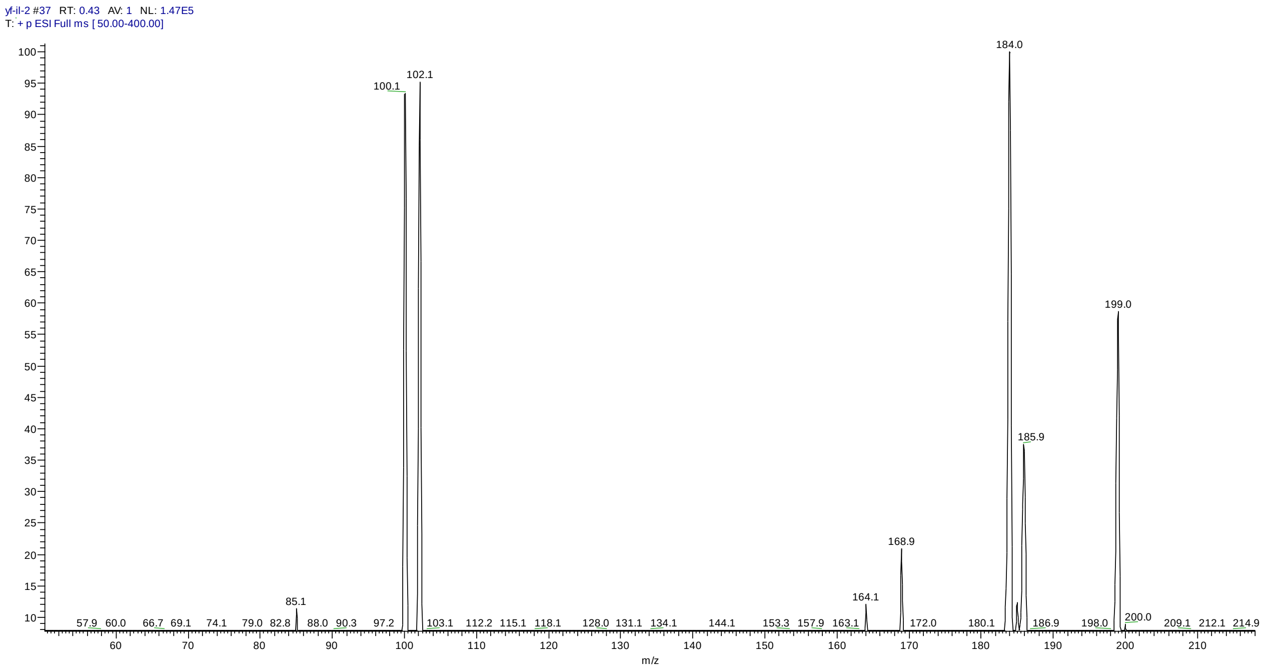

[NMP]PTSA:

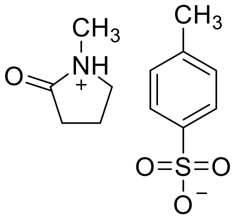

White solid; m. p.: 81-85 ºC; ^{1}H NMR (400 MHz, $CDCl_3$) δ: 2.10-2.17 (m, 2H), 2.36 (s, 3H), 2.86-2.90 (t, J = 8.1 Hz, 2H),2.97 (s, 3H), 3.56-3.60 (t, J = 7.3 Hz, 2H), 7.19-7.26 (m, 2H), 7.74-7.76 (d, J = 8.2 Hz, 2H) ppm; ^{13}C NMR (100 MHz, $CDCl_3$) δ: 17.59, 21.53, 30.39, 31.19, 51.71, 126.30, 129.20, 139.67, 141.68, 178.48 ppm; FT-IR (KBr) Vmax/ cm^{-1}: 3419.96, 2950.36, 2839.67, 1650.69, 1453.04, 1189.09, 1124.96, 1033.54, 1012.40, 817.51, 686.28, 568.77; ESI-MS: m/z (-) 171.1, 268.8.

^{1}H NMR spectrum of [NMP]PTSA:

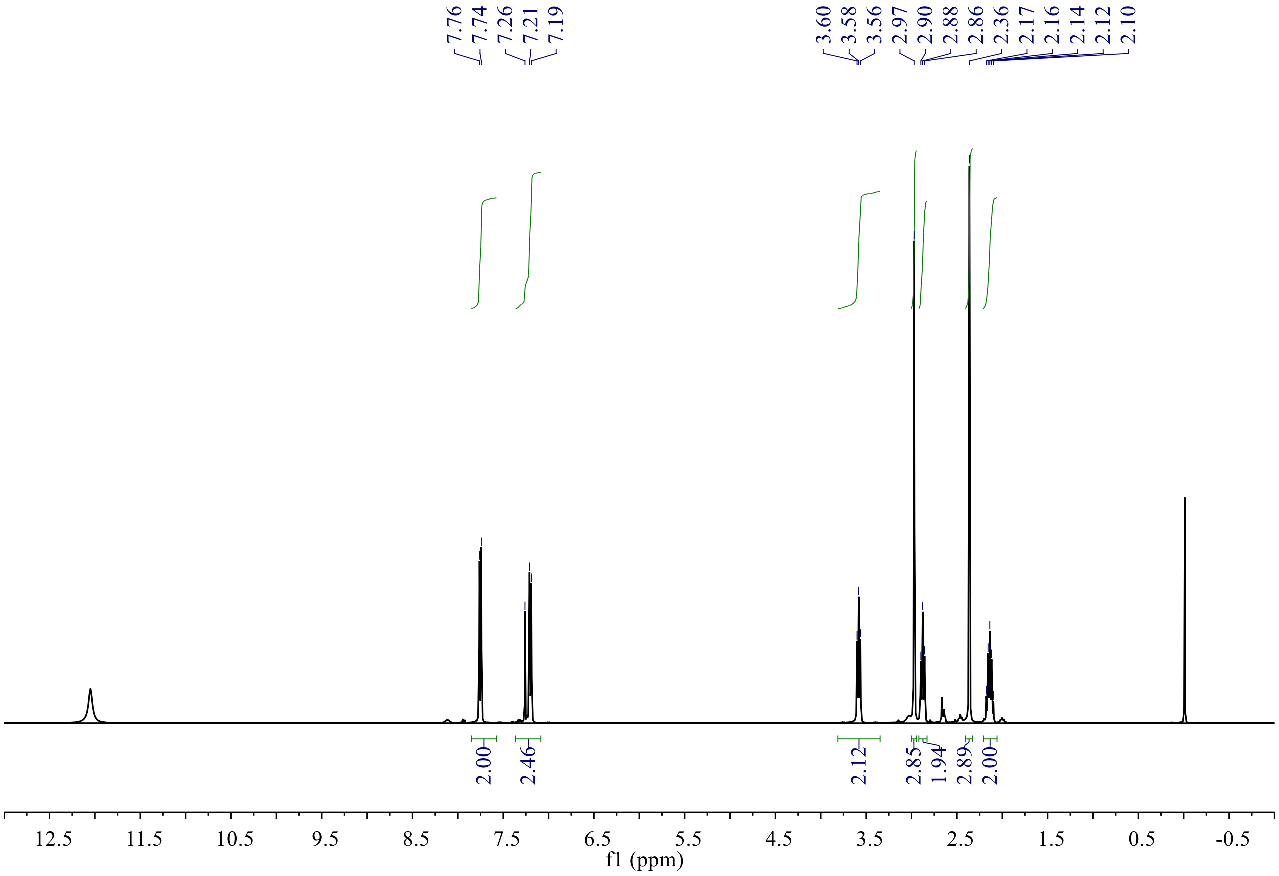

^{13}C NMR spectrum of [NMP]PTSA:

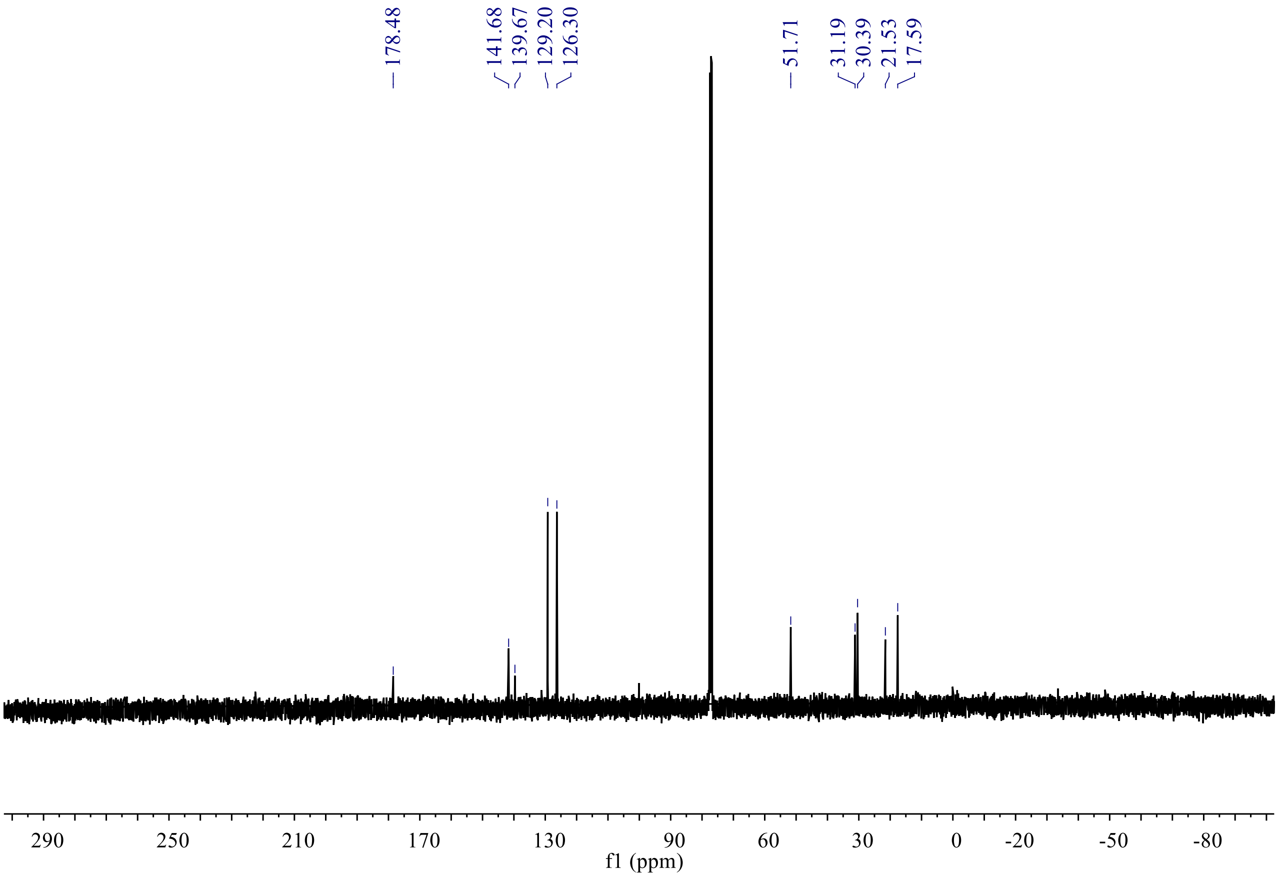

FT-IR spectrum of [NMP]PTSA:

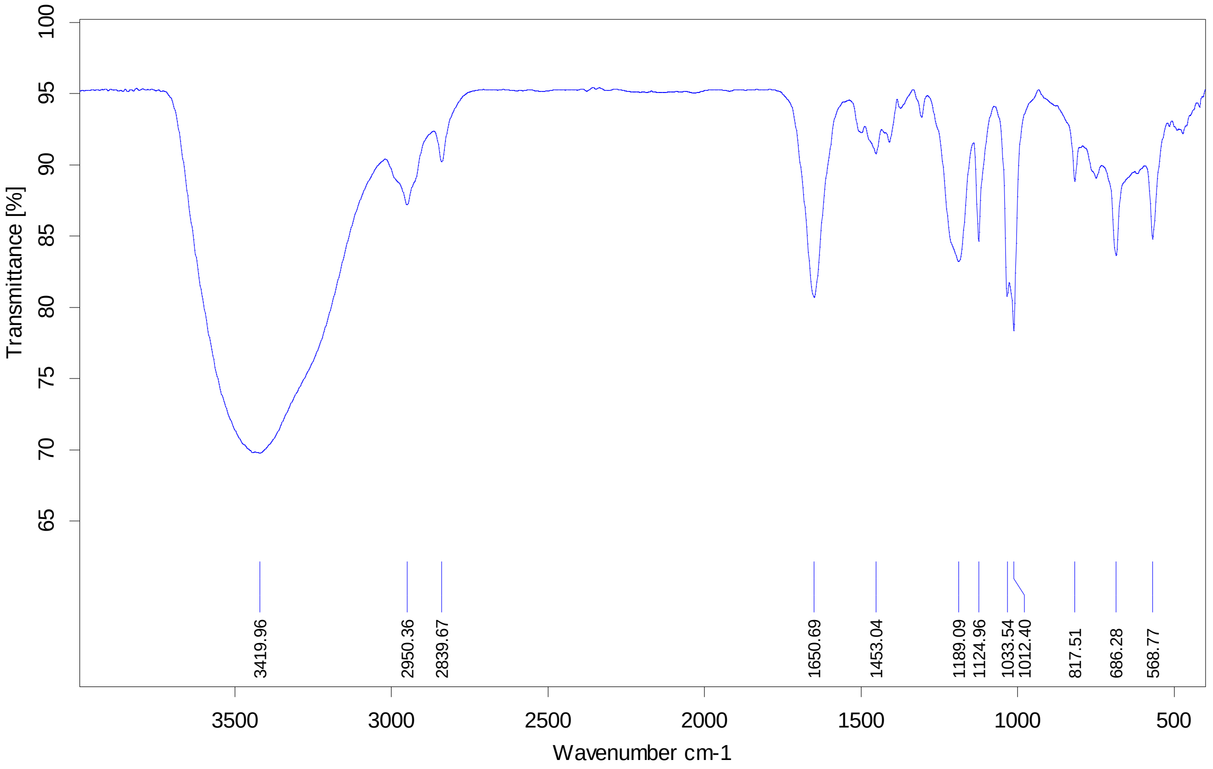

ESI-MS spectrum of [NMP]PTSA:

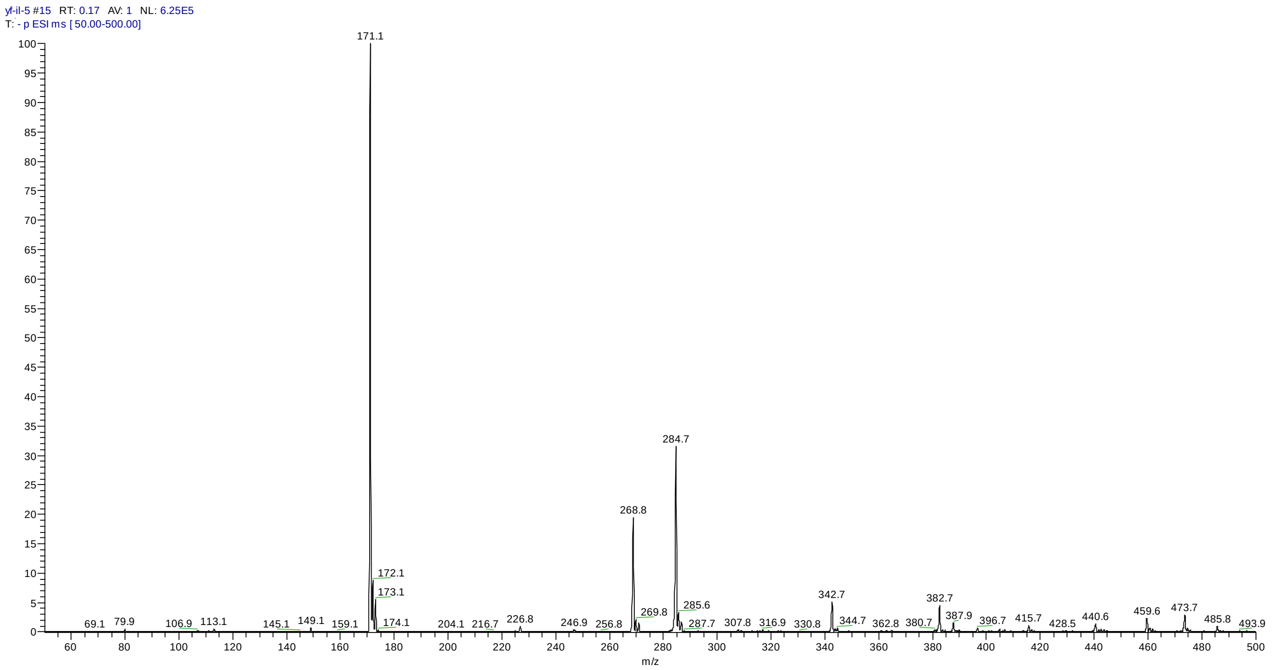

[NMP]CF_3SO_3:

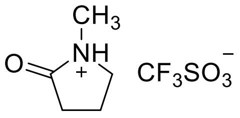

Yellow solid; m. P.: 106-111 ºC; ^{1}H NMR (400 MHz, $CDCl_3$) δ: 2.22-2.30 (m, 2H), 2.98-3.02 (t, J = 8.1 Hz, 2H), 3.10 (s, 3H), 3.77-3.80 (t, J = 7.6 Hz, 2H) ppm; ^{13}C NMR (100 MHz, $CDCl_3$) δ: 17.10, 30.22, 31.72, 53.10, 115.24-124.74, 178.31 ppm; FT-IR (KBr) Vmax/ cm^{-1}: 3424.48, 2953.94, 2924.80, 2849.82, 1697.70, 1495.17, 1460.72, 1253.22, 1169.22, 1031.03, 974.59, 749.83, 639.16, 576.27, 518.11; ESI-MS: m/z (+) 100.1, 183.9, 199.0.

^{1}H NMR spectrum of [NMP]CF_3SO_3:

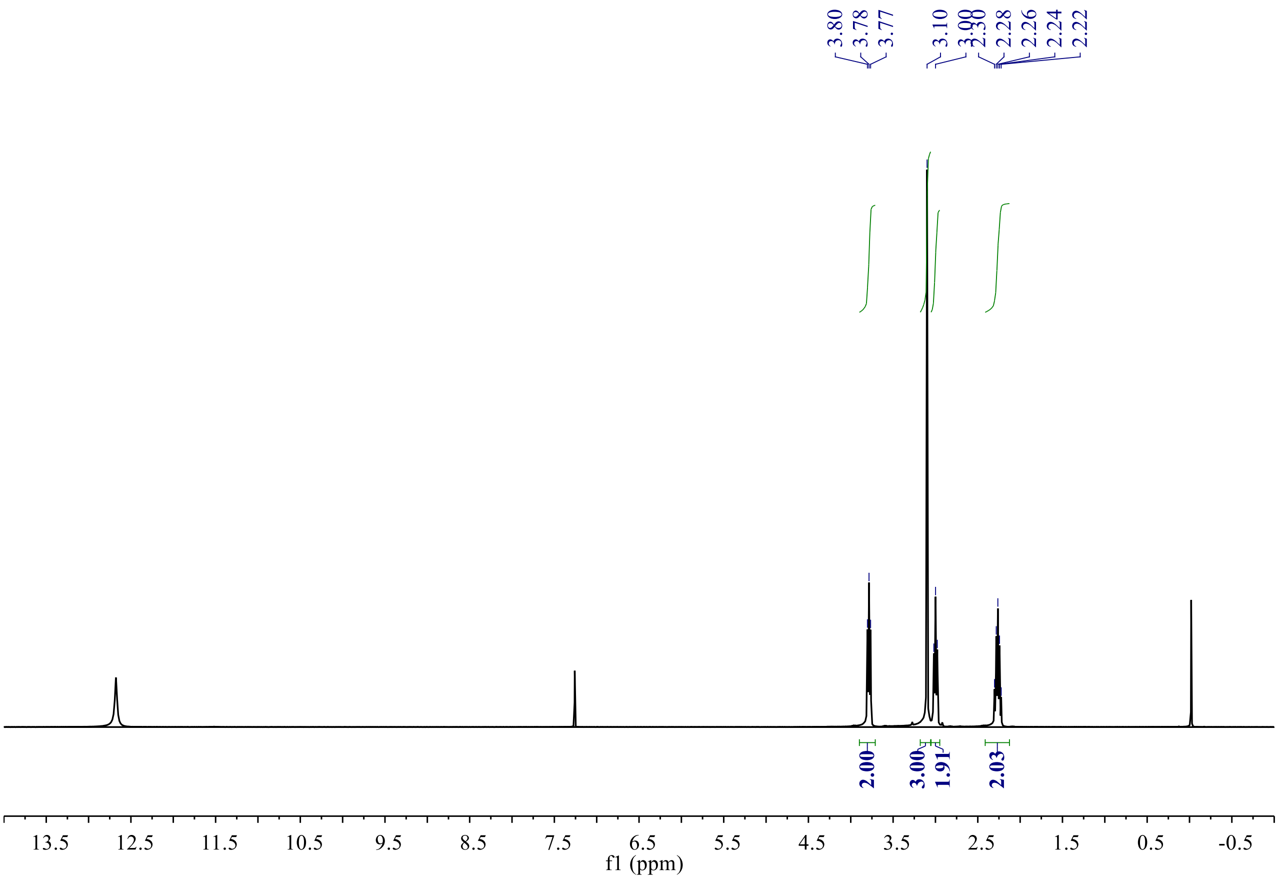

^{13}C NMR spectrum of [NMP]CF_3SO_3:

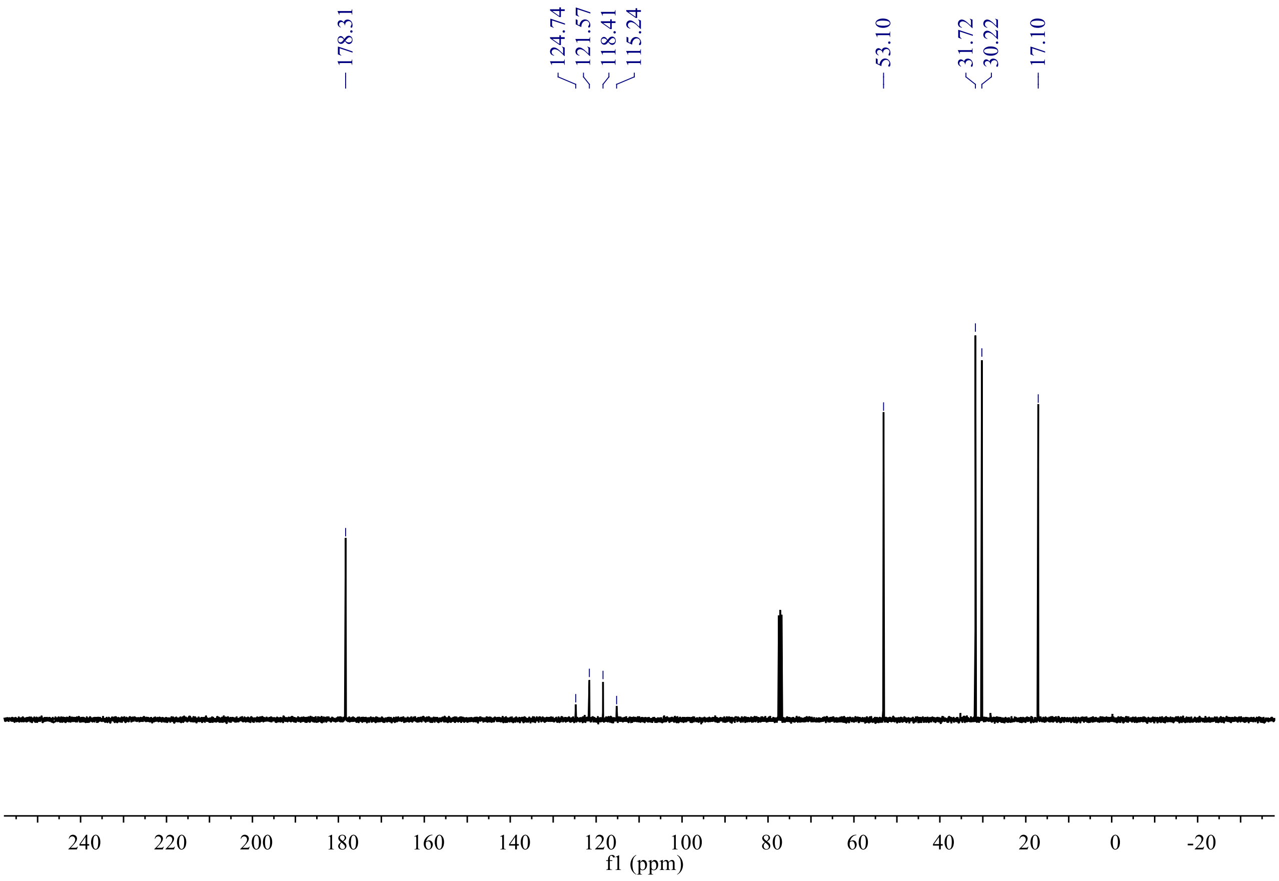

FT-IR spectrum of [NMP]CF_3SO_3:

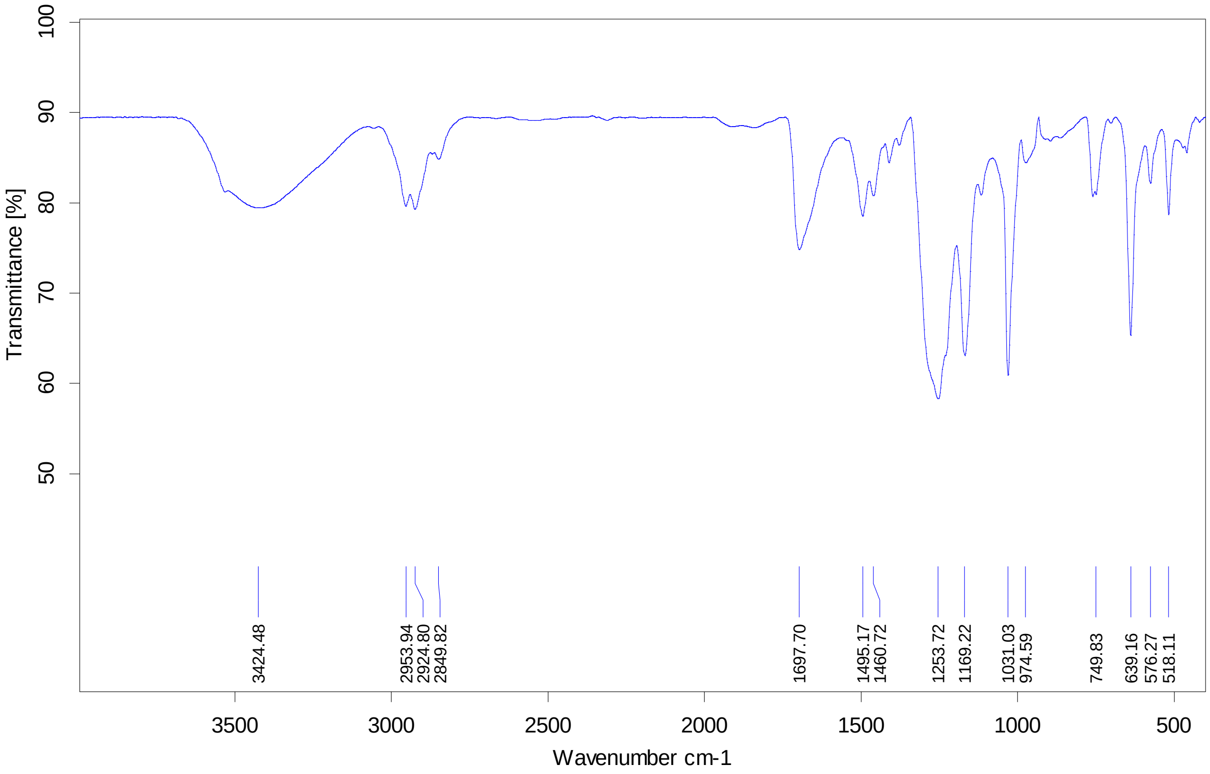

ESI-MS spectrum of [NMP]CF_3SO_3:

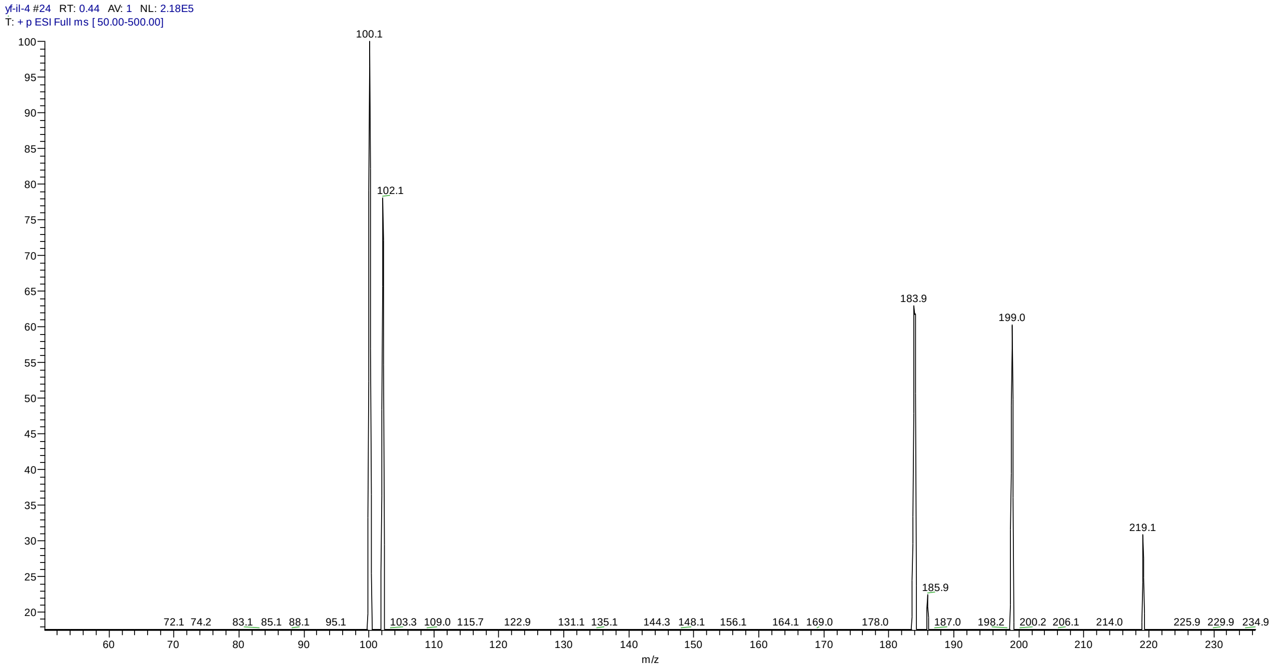

[HSO_3-BDBU]H_2PO_4:

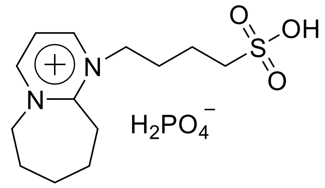

Yellow viscous liquid; ^{1}H NMR (400 MHz, DMSO-d_6) δ: 1.62-1.67 (m, 10H), 1.92-1.98 (m, 2H), 2.41-2.45 (t, J = 6.8 Hz, 2H), 2.85-2.88 (t, J = 4.8, 2H), 3.42-3.62 (m, 8H) ppm; ^{13}C NMR (100 MHz, DMSO-d_6) δ: 19.65, 22.13, 22.88, 25.55, 27.12, 27.22, 27.90, 46.58, 48.52, 50.65, 52.92, 53.98, 165.98 ppm; FT-IR (KBr) Vmax/ cm^{-1}: 3317.18, 2939.11, 2867.62, 1621.52, 1527.51, 1452.10, 1328.75, 1201.00, 998.74, 726.90, 600.28; ESI-MS: m/z (+) 100.1, 102.1, 153.2, 289.3, 390.1.

^{1}H NMR spectrum of [HSO_3-BDBU]H_2PO_4:

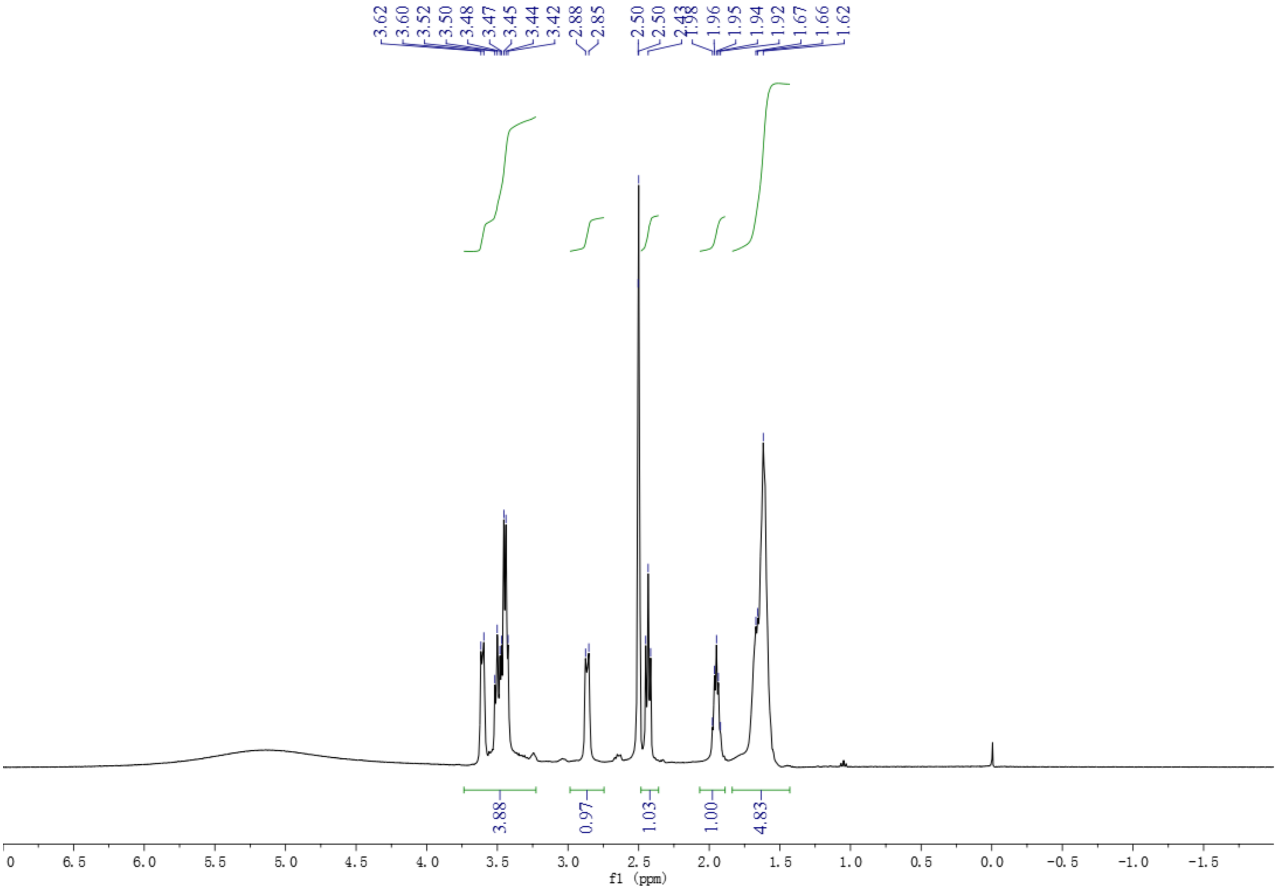

^{13}C NMR spectrum of [HSO_3-BDBU]H_2PO_4:

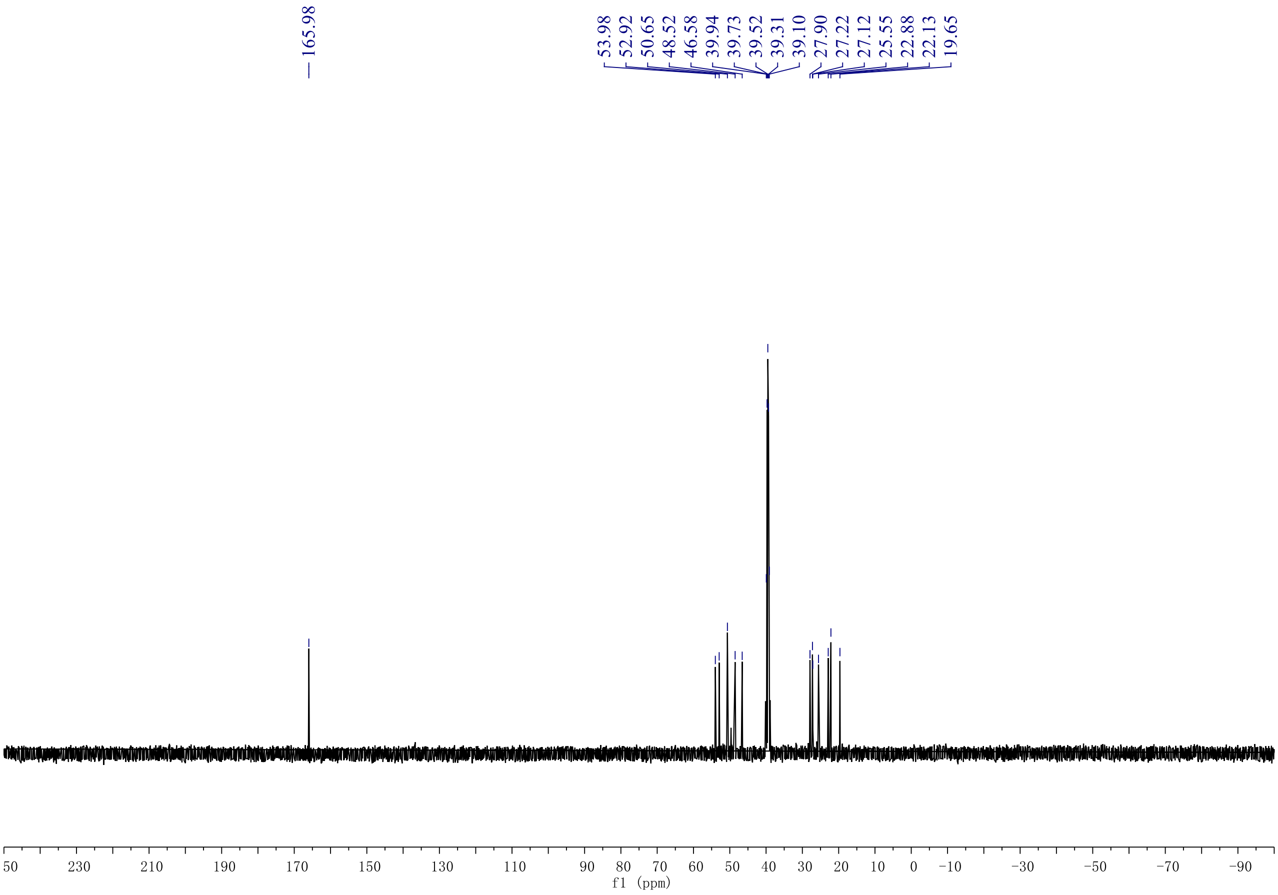

FT-IR spectrum of $[HSO_3\text{-}BDBU]H_2PO_4$:

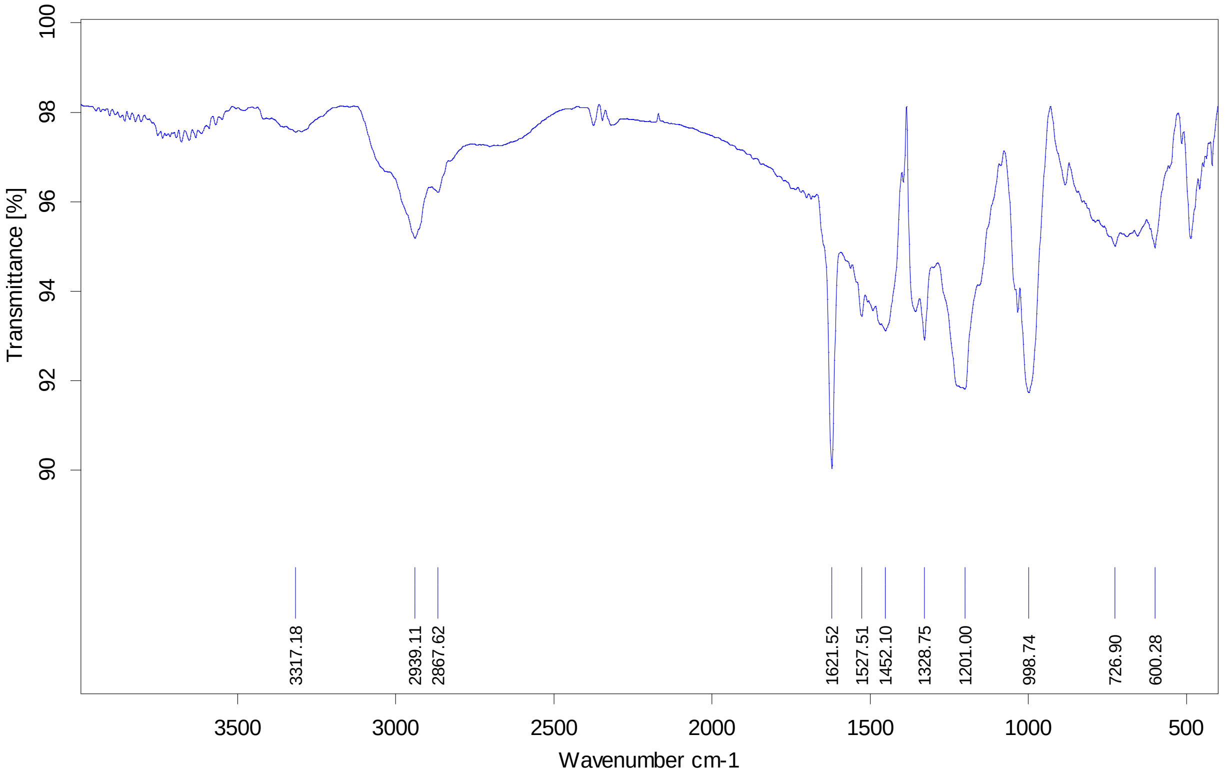

ESI-MS spectrum of $[HSO_3\text{-}BDBU]H_2PO_4$:

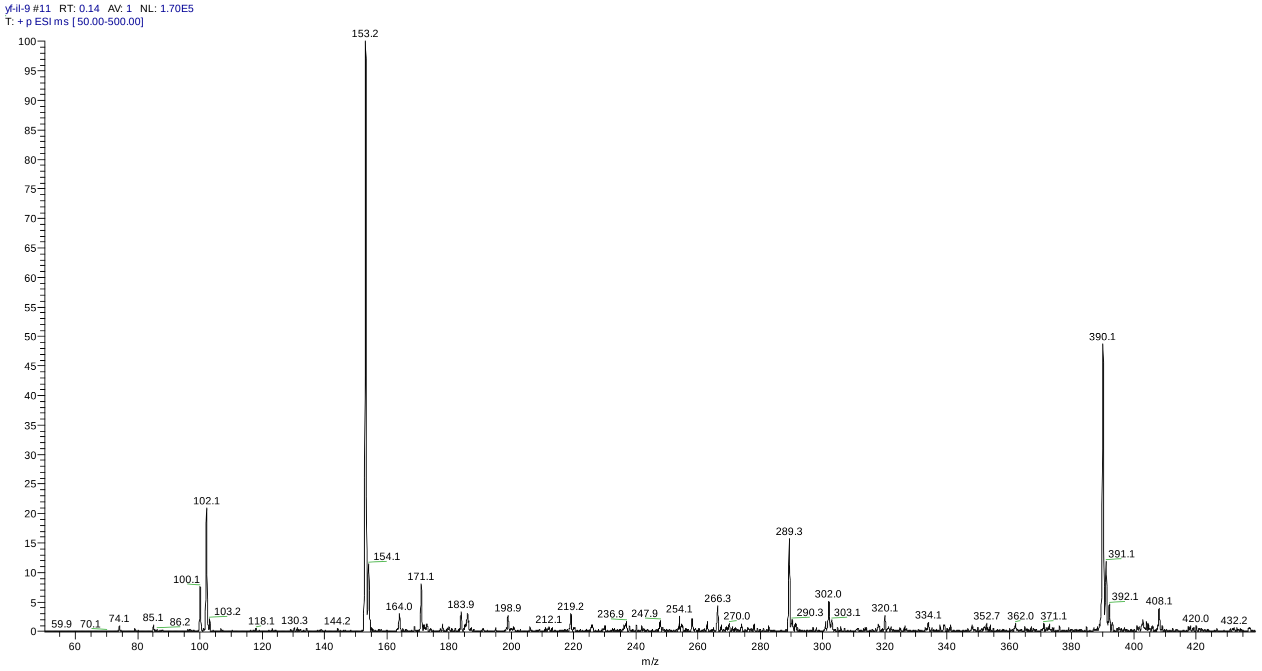

[HSO3-BPy]H2PO4:

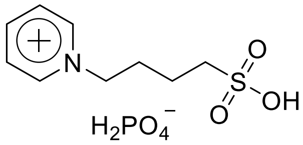

Colorless viscous liquid; ^{1}H NMR (400 MHz, D_2O) δ: 1.77-1.85 (m, 2H), 2.15-2.22 (m, 2H), 2.95-2.99 (t, J = 7.6 Hz, 2H), 4.65-4.69 (t, J = 7.6 Hz, 2H), 8.06-8.10 (t, J = 6.9 Hz, 2H), 8.53-8.57 (t, J = 7.8 Hz, 1H), 8.86-8.88 (d, J = 6.0 Hz, 2H) ppm; ^{13}C NMR (100 MHz, D_2O) δ: 20.84, 29.28, 49.91, 61.18, 128.28, 144.23, 145.65 ppm; FT-IR (KBr) *V*max/ cm^{-1}: 3418.43, 2950.08, 2843.38, 2308.70, 1637.56, 1490.61, 1176.73, 1004.85, 886.76, 768.27, 681.73, 486.51; ESI-MS: m/z (+) 100.1, 102.1, 317.0.

^{1}H NMR spectrum of [HSO3-BPy]H2PO4:

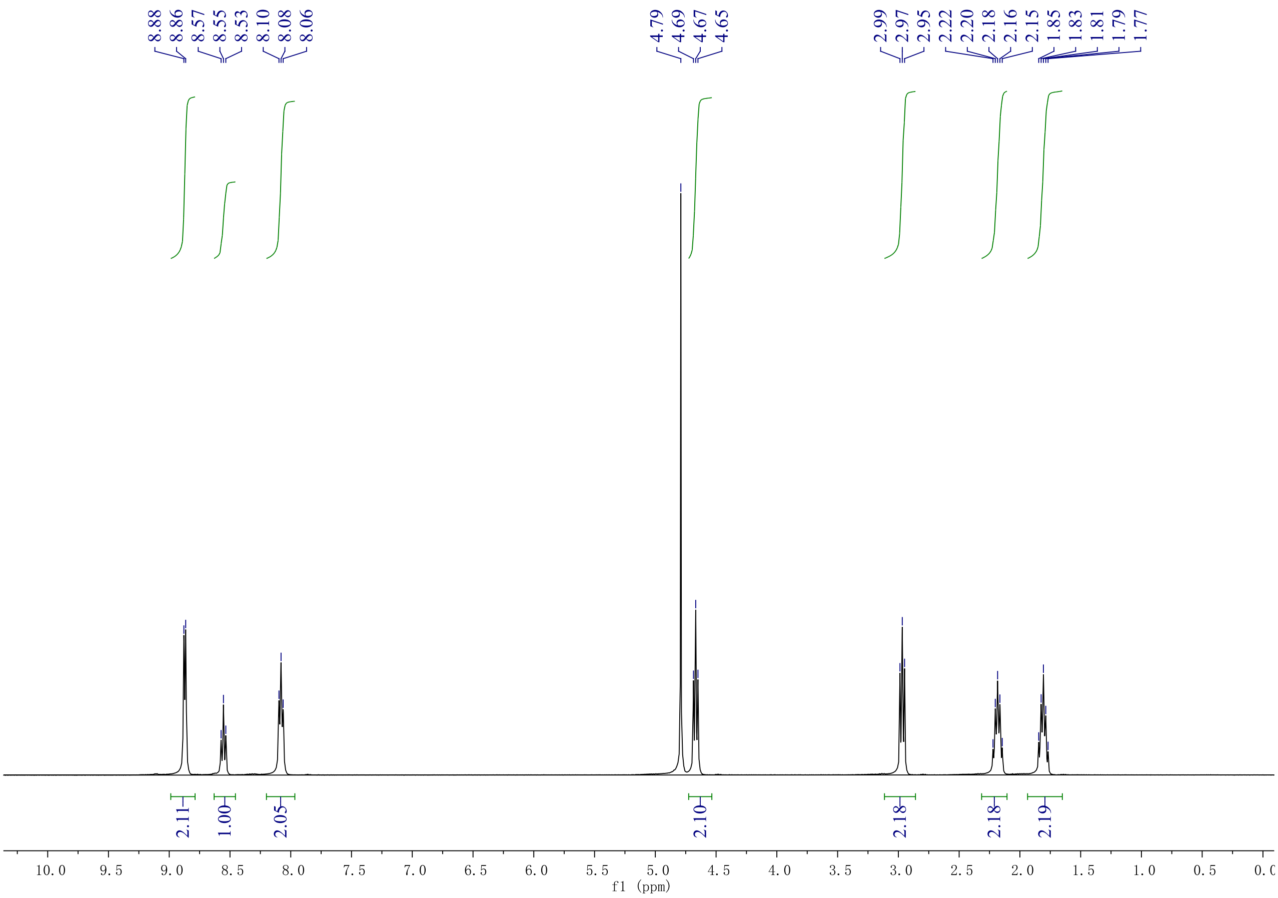

^{13}C NMR spectrum of $[HSO_3\text{-}BPy]H_2PO_4$:

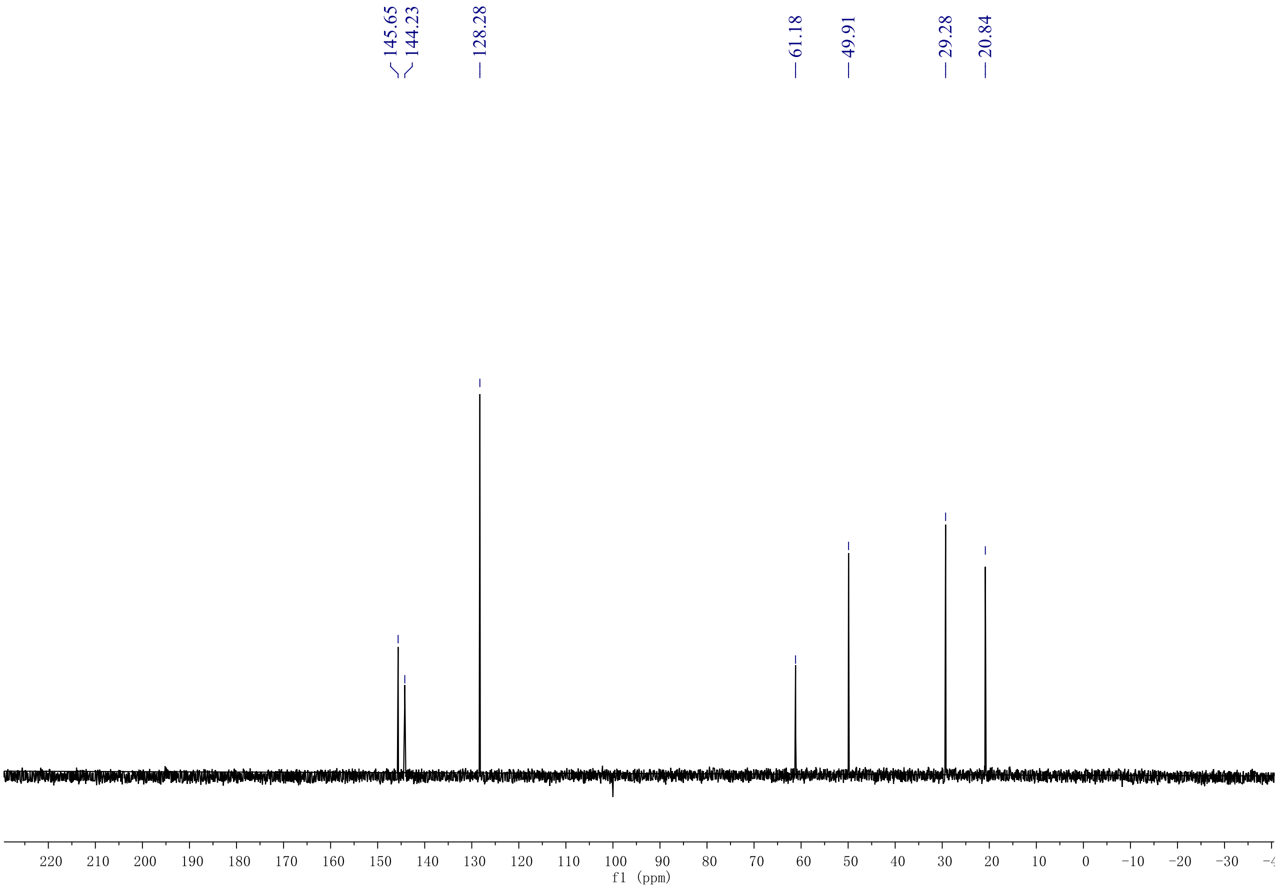

FT-IR spectrum of $[HSO_3\text{-}BPy]H_2PO_4$:

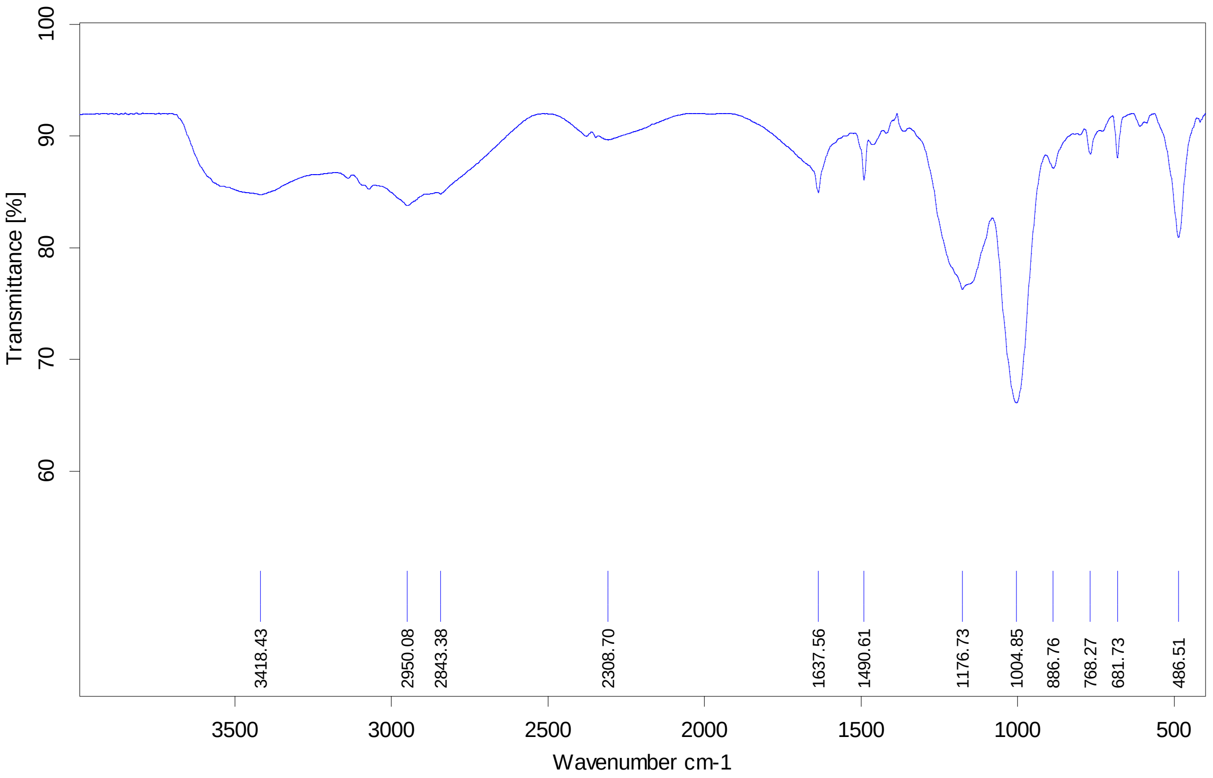

ESI-MS spectrum of $[HSO_3\text{-}BPy]H_2PO_4$:

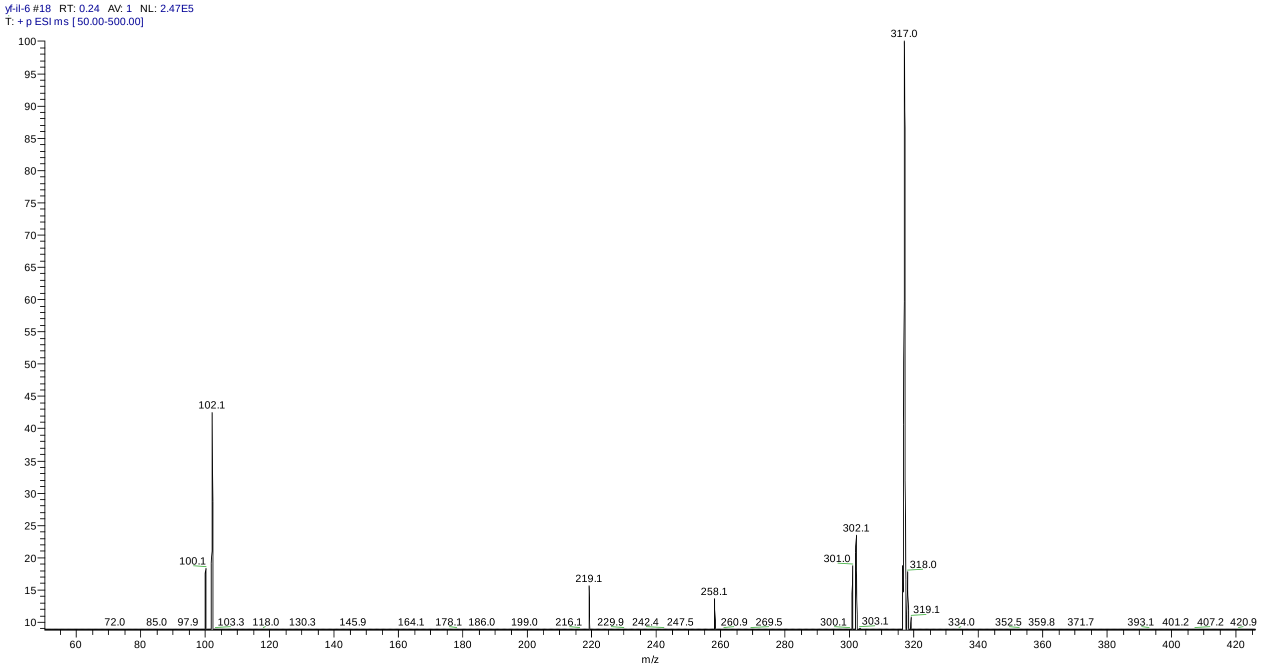

[HSO3-BMim]H2PO4:

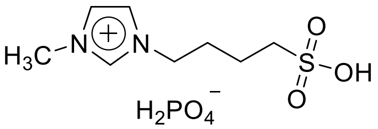

Colorless viscous liquid; ^{1}H NMR (400 MHz, D_2O) δ: 1.66-1.74 (m, 2H), 1.95-2.02 (m, 2H), 2.89-2.92 (t, J = 7.6 Hz, 2H), 3.85 (s, 3H), 4.19-4.23 (t, J = 7.2 Hz, 2H), 7.40-7.46 (2H), 8.71 (s, 1H) ppm; ^{13}C NMR (100 MHz, D_2O) δ: 20.59, 27.76, 35.37, 48.60, 49.74, 121.85, 123.35, 135.57 ppm; FT-IR (KBr) Vmax/ cm^{-1}: 3161.71, 2938.26, 2308.85, 1573.27, 1460.51, 1167.84, 1001.78, 886.43, 745.78, 484.87; ESI-MS: m/z (+) 100.1, 102.1, 219.3, 320.1.

^{1}H NMR spectrum of [HSO3-BMim]H2PO4:

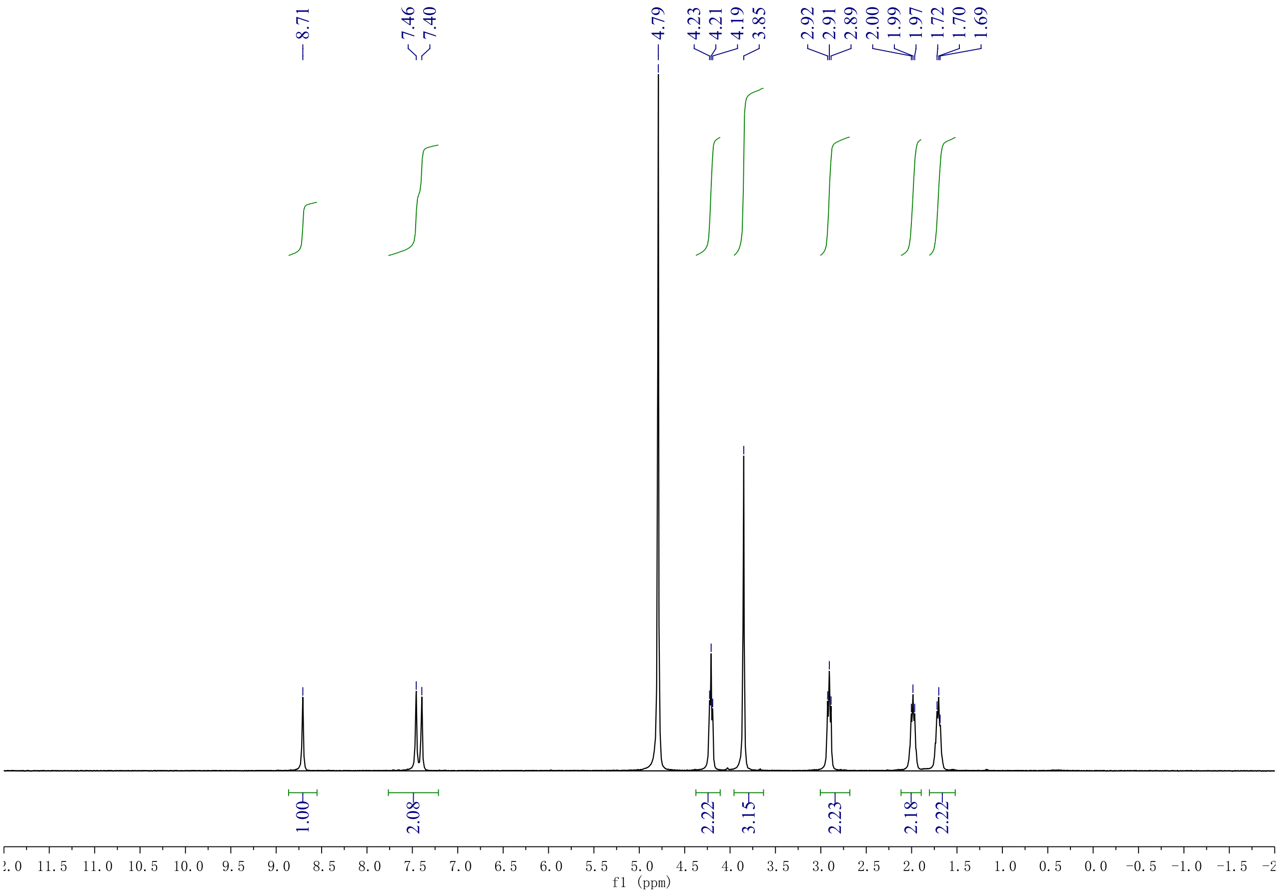

^{13}C NMR spectrum of [HSO_3-BMim]H_2PO_4:

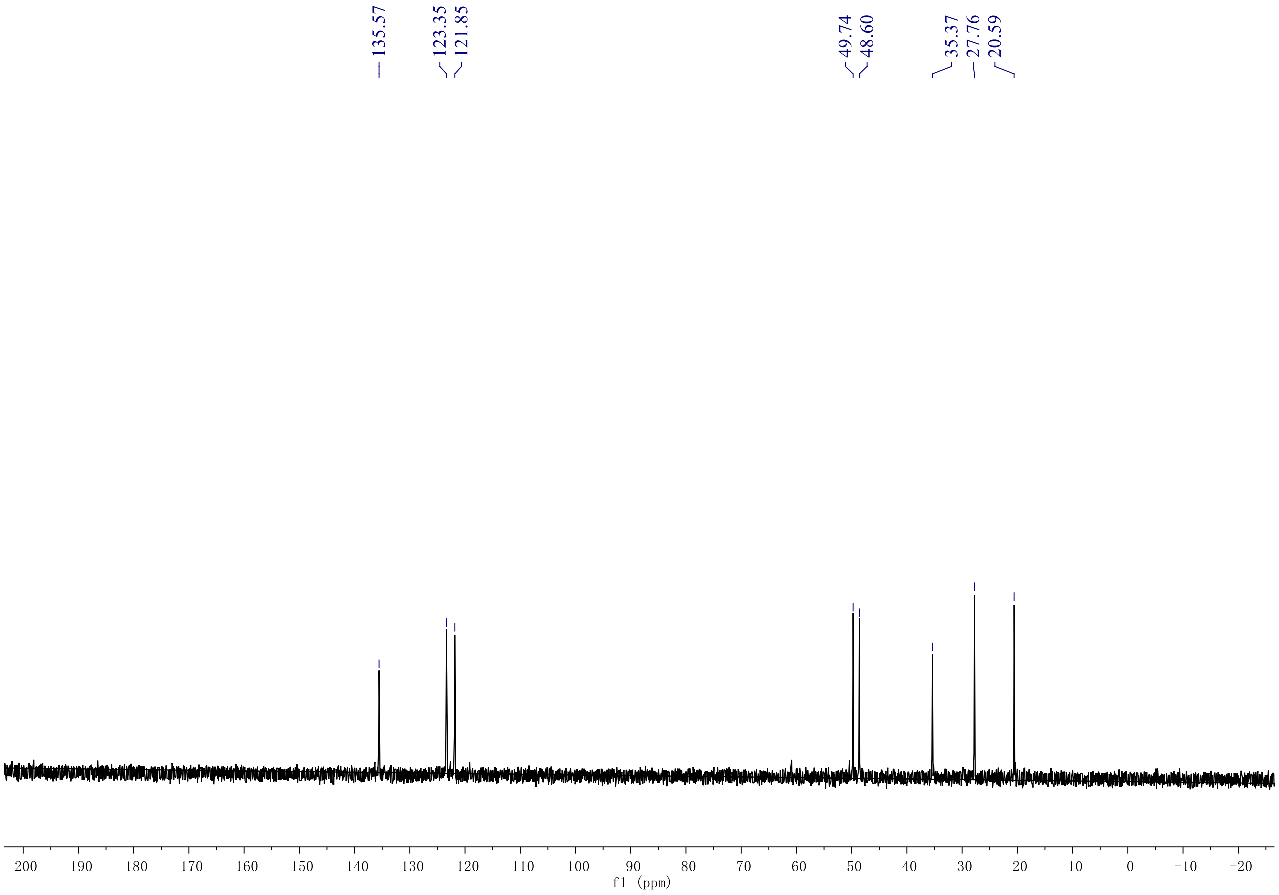

FT-IR spectrum of [HSO_3-BMim]H_2PO_4:

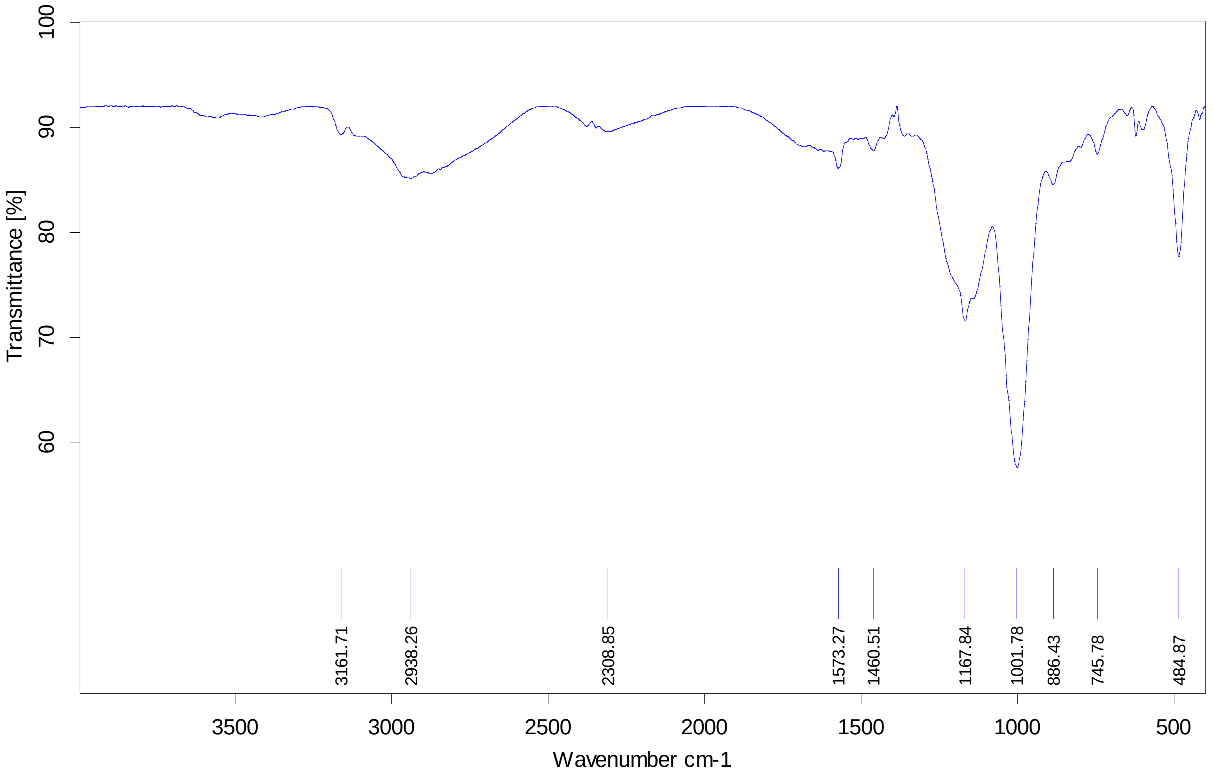

ESI-MS spectrum of [HSO_3-BMim]H_2PO_4:

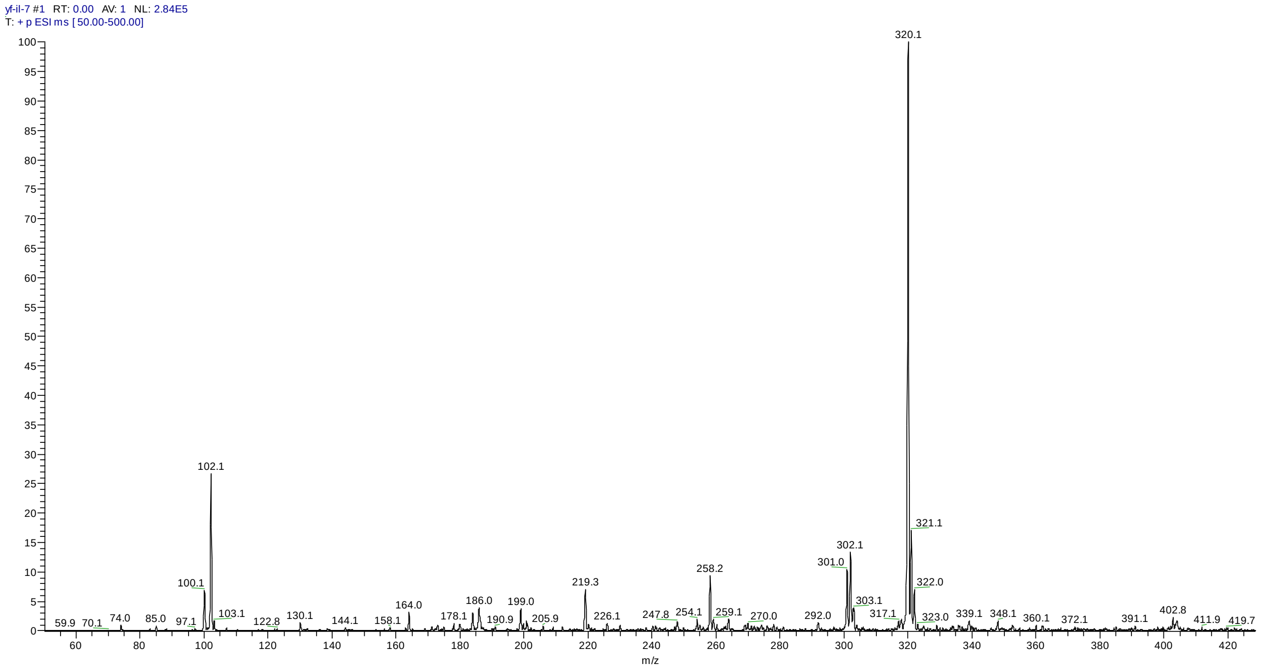

[HSO3-BNEt3]H2PO4:

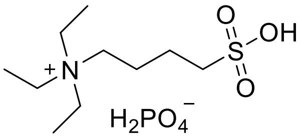

Colorless viscous liquid; ^{1}H NMR (400 MHz, D_2O) δ: 1.22-1.26 (t, J = 6.8, 9H), 1.77-1.83 (m, 4H), 2.92-2.96 (t, J = 6.8 Hz, 2H), 3.17-3.29 (m, 8H) ppm; ^{13}C NMR (100 MHz, D_2O) δ: 6.55, 19.86, 21.16, 49.96, 52.53, 55.90 ppm; FT-IR (KBr) *V*max/ cm^{-1}: 3679.26, 2950.42, 1487.43, 1361.83, 1213.30, 1004.20, 884.39, 799.40, 730.67, 608.27 488.00; ESI-MS: m/z (+) 100.1, 102.1, 238.3, 339.1.

^{1}H NMR spectrum of [HSO3-BNEt3]H2PO4:

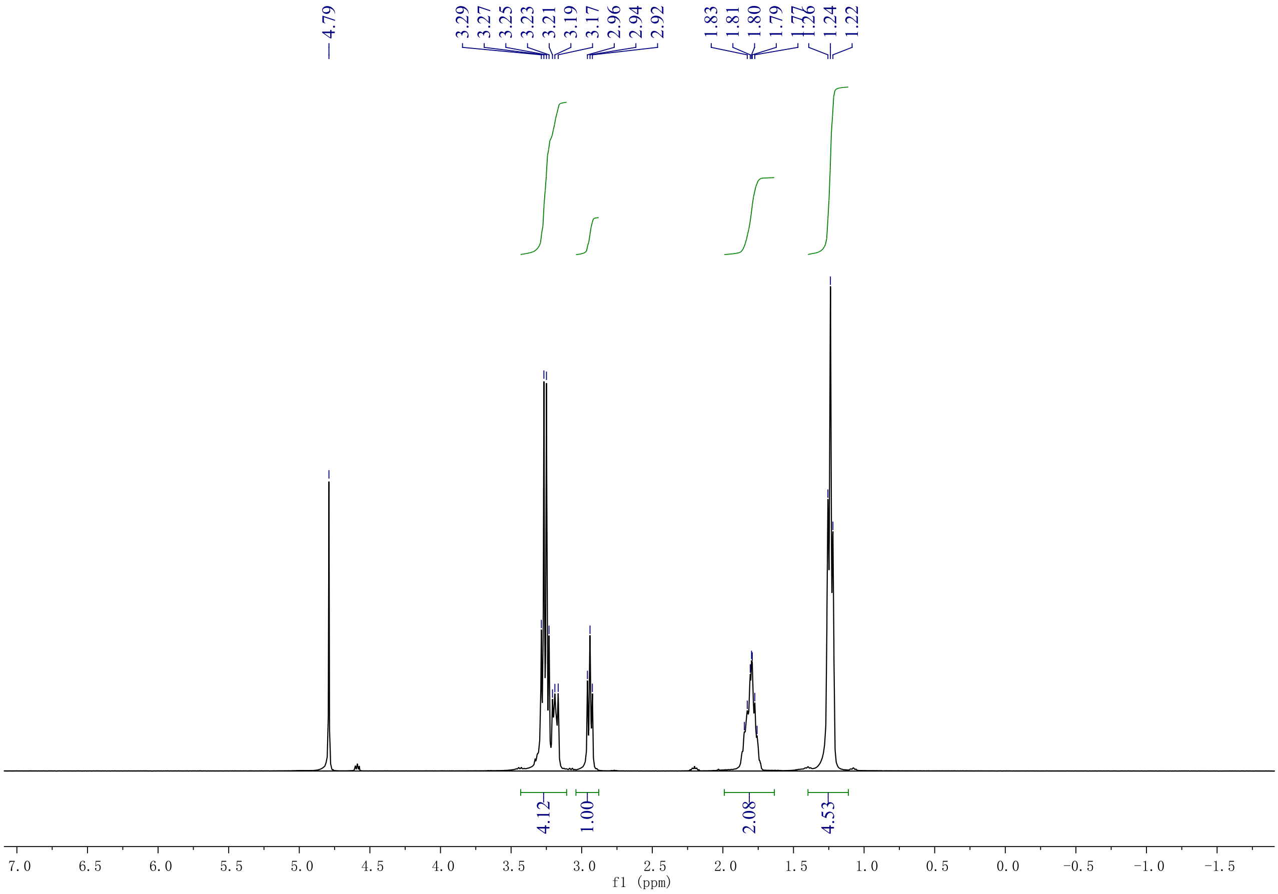

^{13}C NMR spectrum of $[HSO_3\text{-}BNEt_3]H_2PO_4$:

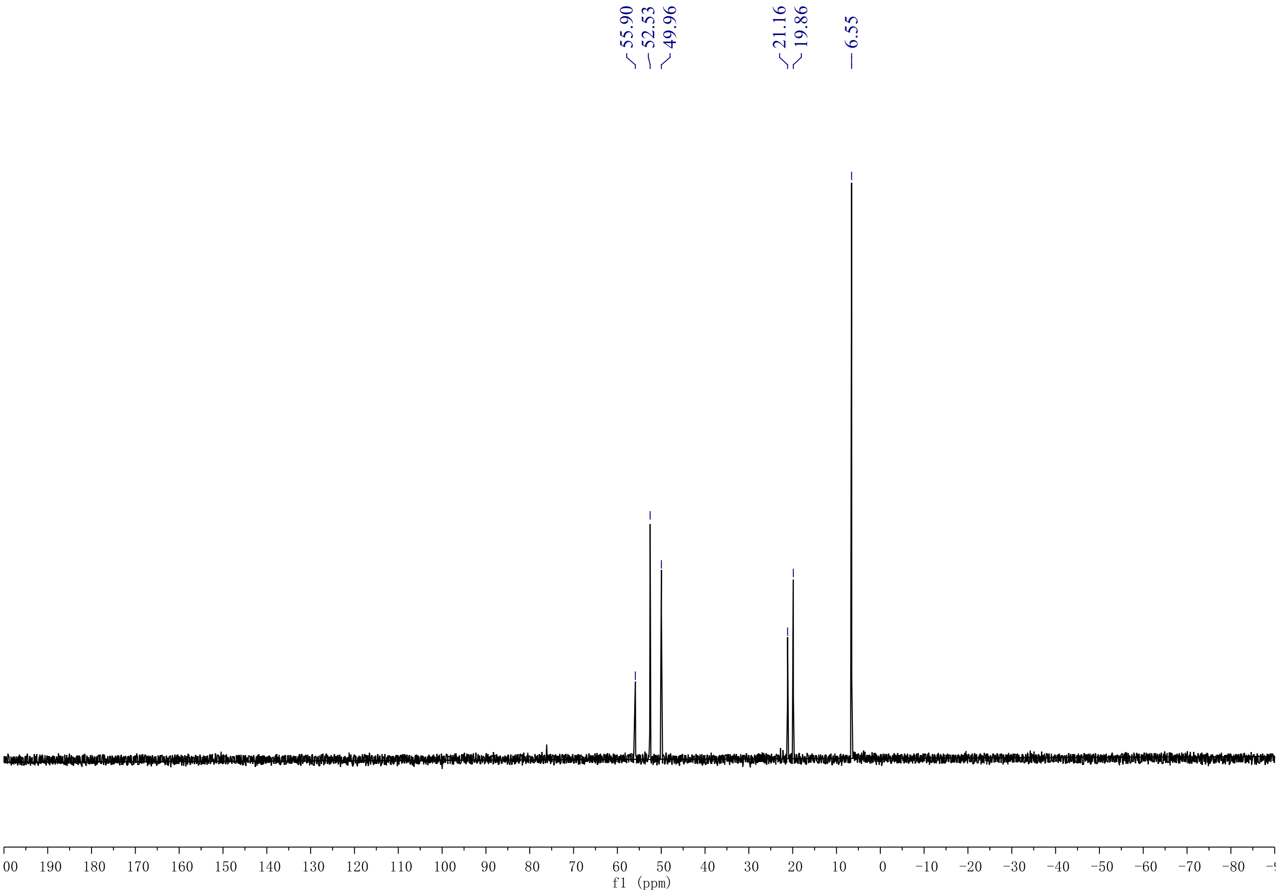

FT-IR spectrum of $[HSO_3\text{-}BNEt_3]H_2PO_4$:

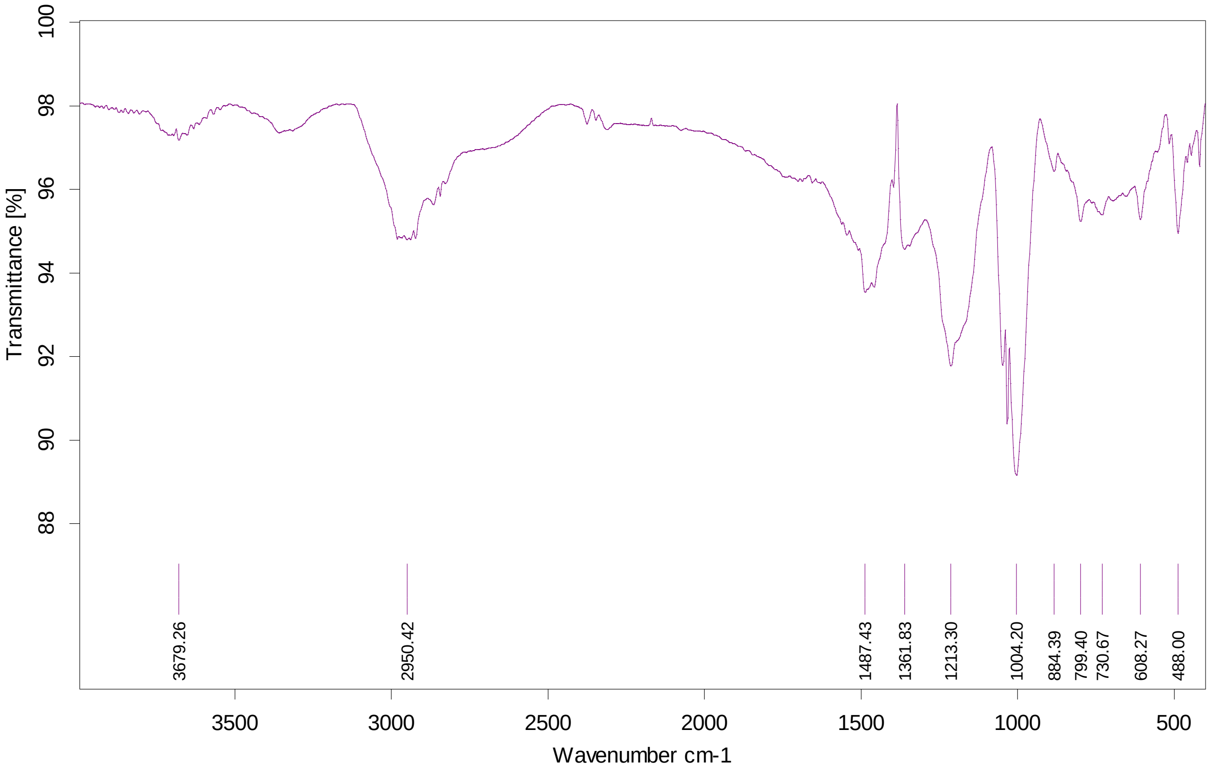

ESI-MS spectrum of $[HSO_3\text{-}BNEt_3]H_2PO_4$:

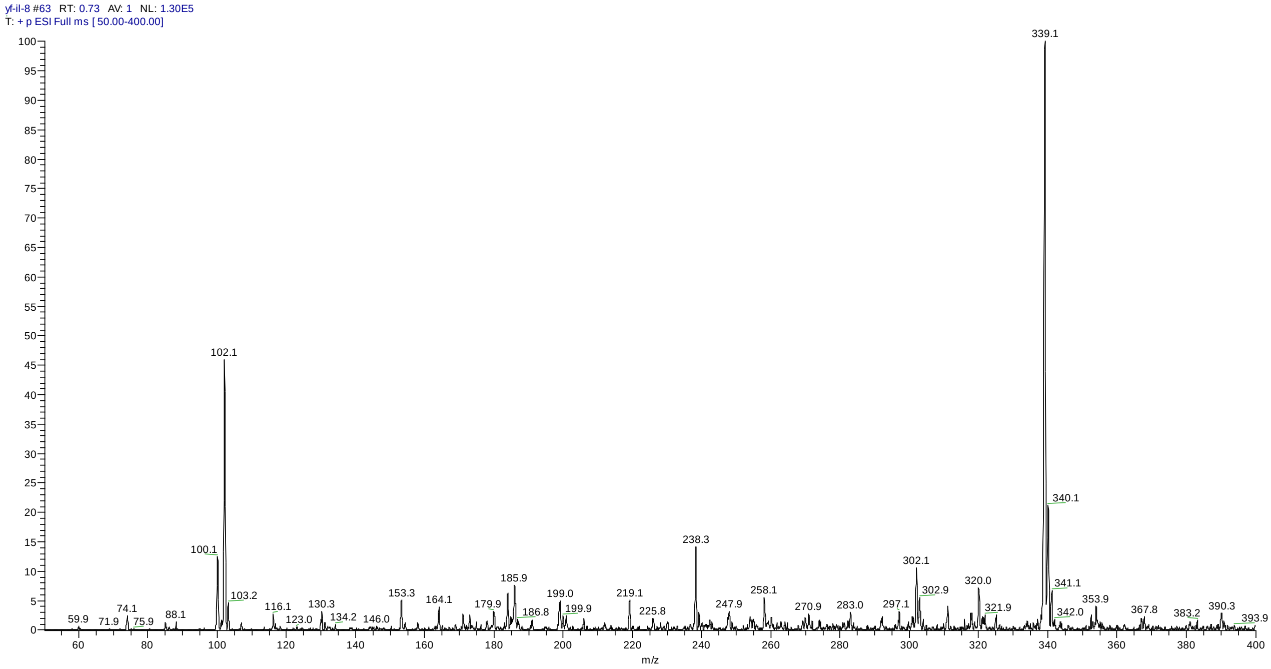

Products characterization

Polyricinoleic acid:

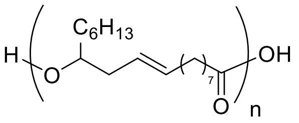

Yellow oily liquid; ^{1}H NMR (400 MHz, $CDCl_3$) δ: 0.85-0.88 (t, J = 3.9 Hz, 3H), 1.26-1.29 (m, 16H), 1.51-1.60 (m, 4H), 2.00-2.01 (m, 2H), 2.25-2.26 (m, 4H), 4.86-4.89 (m, 1H), 5.30-5.46 (m, 2H) ppm; ^{13}C NMR (100 MHz, $CDCl_3$) δ: 14.08, 22.58, 25.11, 25.35, 27.20-27.35, 29.03-29.71, 31.75, 31.98, 33.62, 34.65, 73.69, 124.30, 132.51, 173.58 ppm; FT-IR (KBr) *V*max/ cm^{-1}: 3416.44, 3010.55, 2927.89, 2855.81, 1733.38, 1711.66, 1464.22, 1245.41, 1183.74, 725.11; ESI-MS: m/z (+) 579.3, 876.6, 1139.7, 1437.8, 1716.9, 1997.1.

^{1}H NMR spectrum of polyricinoleic acid:

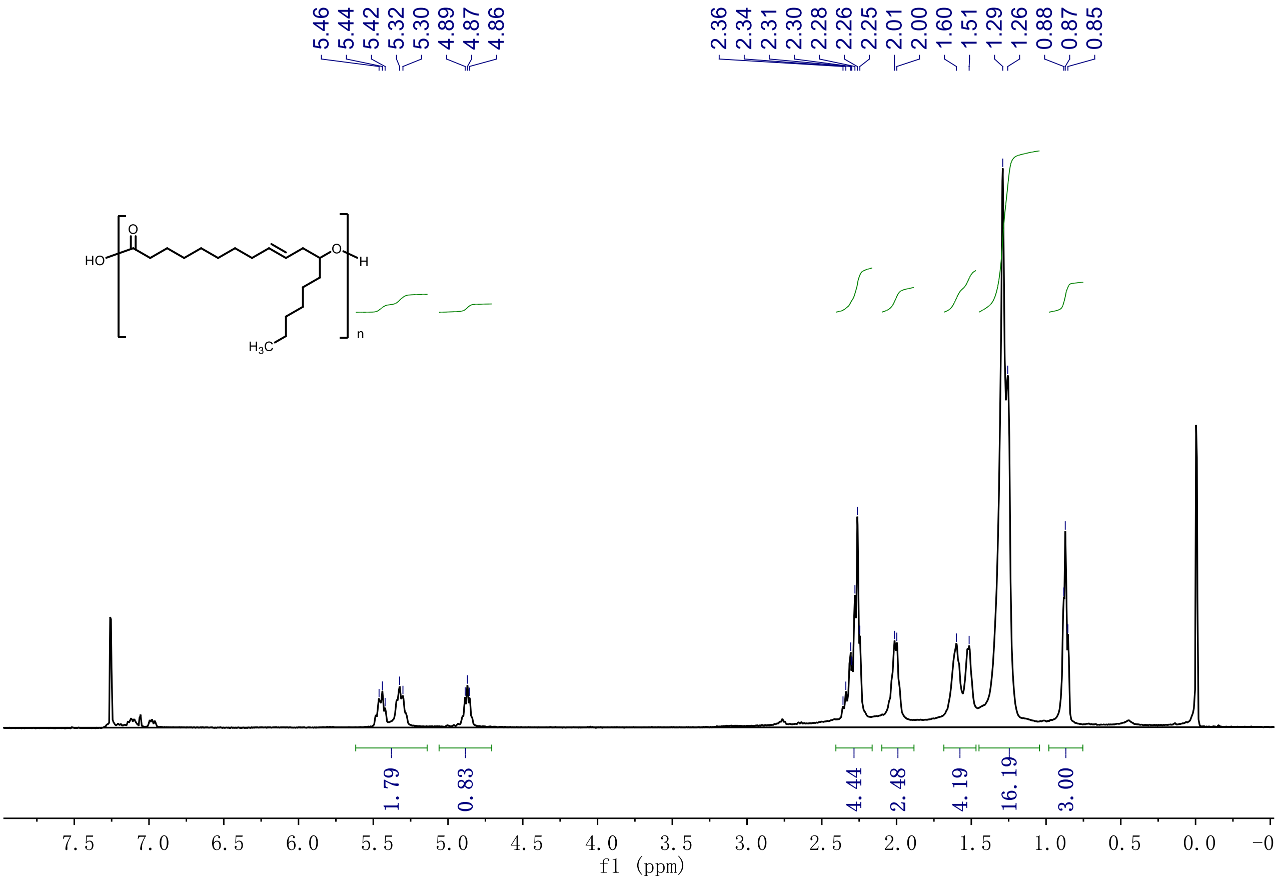

^{13}C NMR spectrum of polyricinoleic acid:

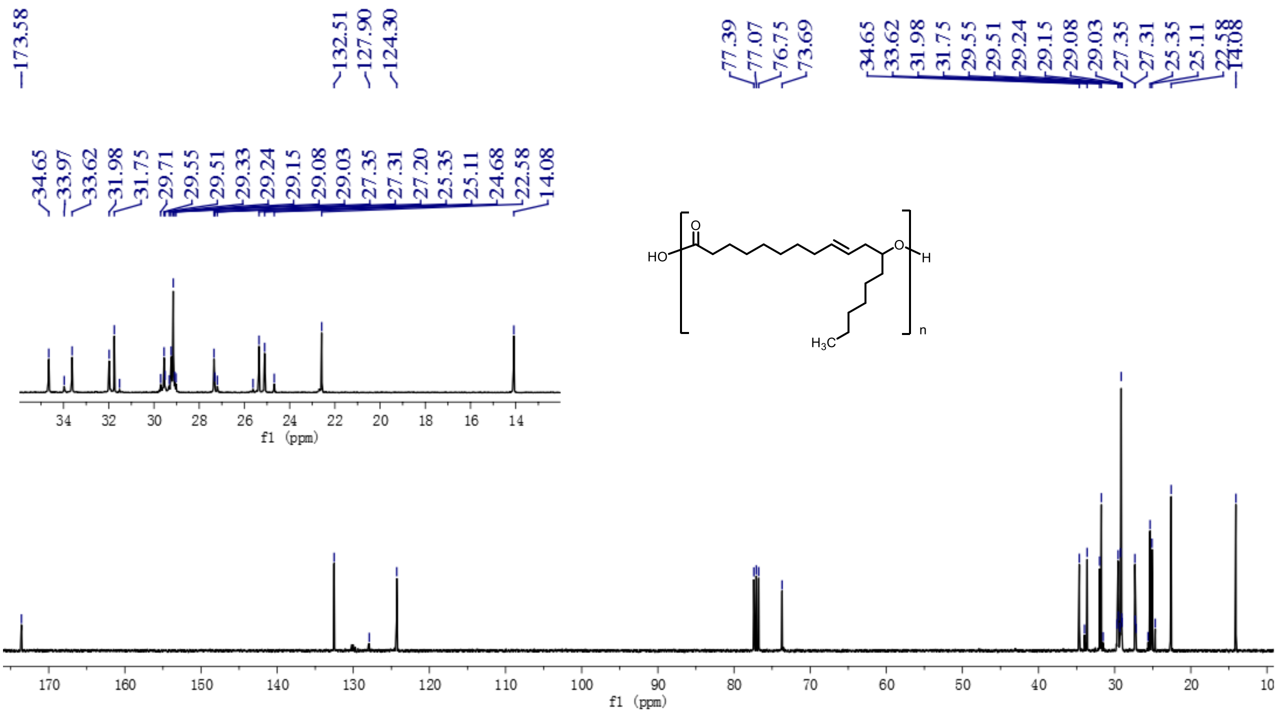

FT-IR spectrum of polyricinoleic acid:

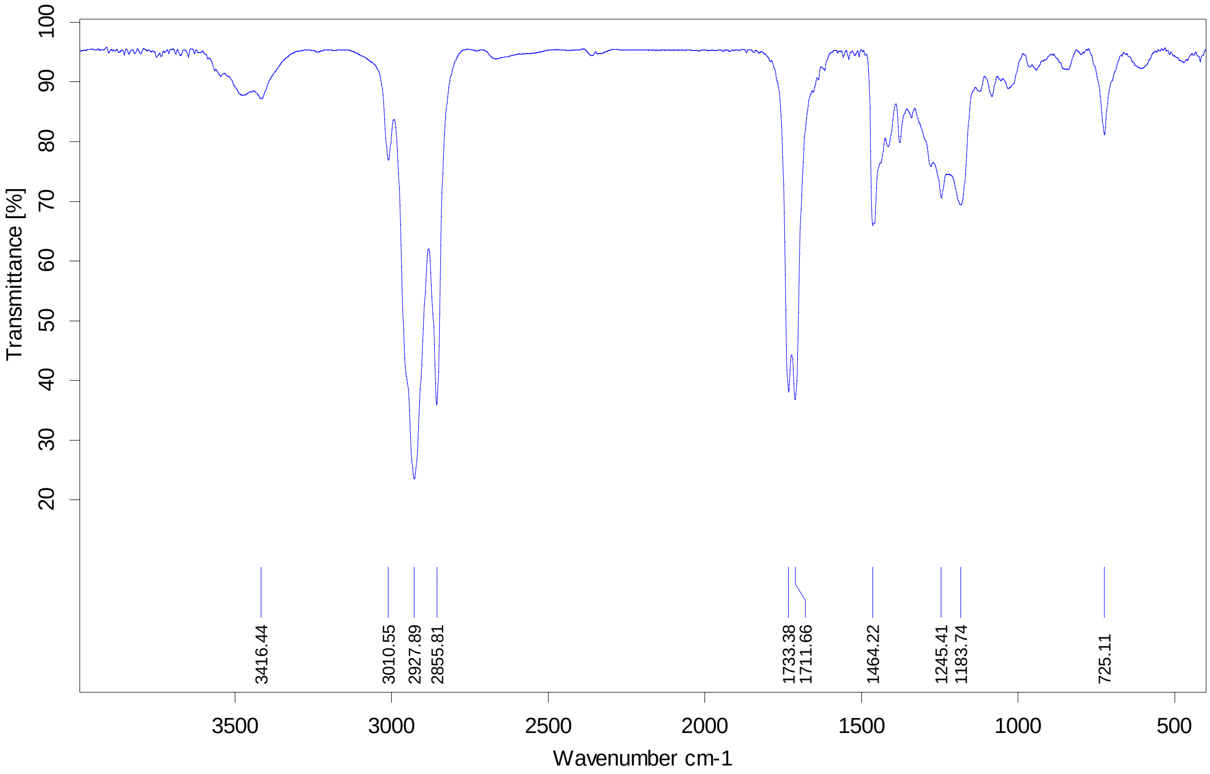

ESI-MS spectrum of polyricinoleic acid:

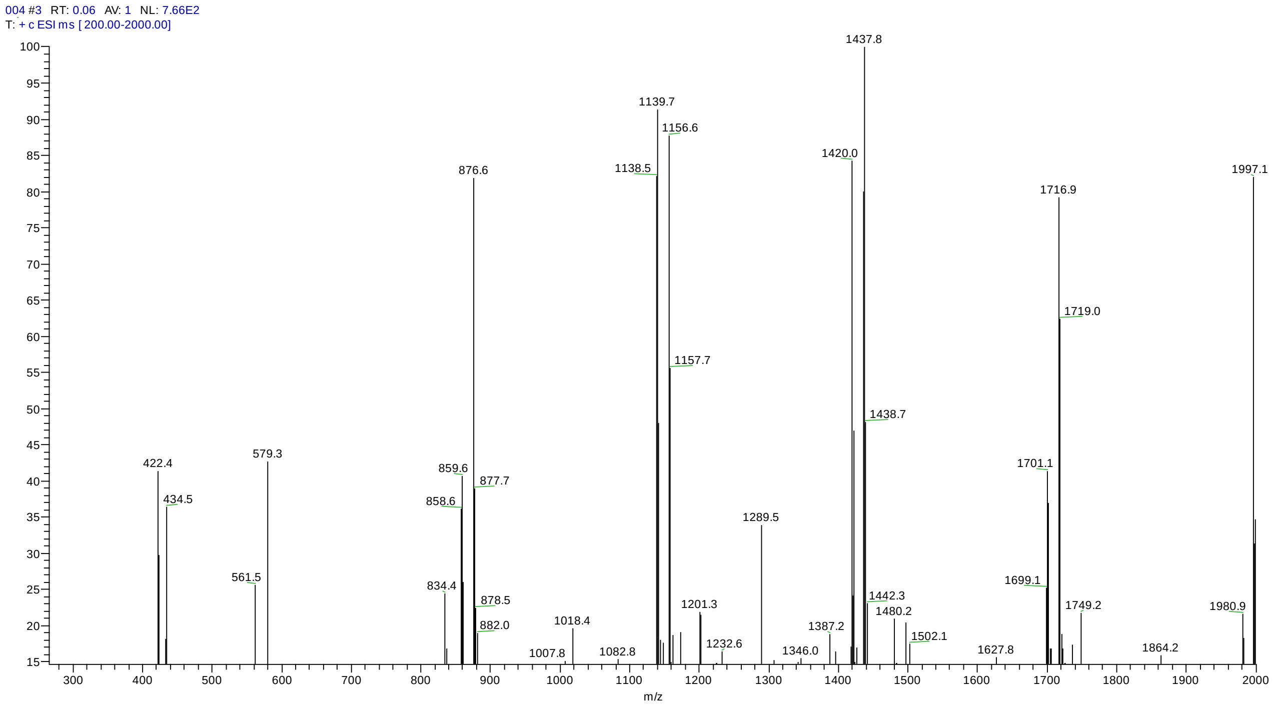

References

[1] Guo, H.; Li, X.; Wang, J. L.;Jin, X. H.; Lin, X. F. Acidic ionic liquid [NMP]H_2PO_4 as dual solvent-catalyst for synthesis of *β*-alkoxyketones by the oxa-Michael addition reactions. *Tetrahedron* **2010**, 66, 8300-8303.

[2] Tao, F. R.; Zhuang, C.; Cui, Y. Z.; Xu, J. Dehydration of glucose into 5-hydroxymethylfurfural in SO_3H-functionalized ionic liquids. *Chinese Chemical Letters* **2014,** *25*, 757-761.
